# *In vivo* synaptic transmission and morphology in mouse models of Tuberous sclerosis, Fragile X syndrome, Neurofibromatosis type 1, and Costello syndrome

**DOI:** 10.3389/fncel.2015.00234

**Published:** 2015-07-03

**Authors:** Tiantian Wang, Laura de Kok, Rob Willemsen, Ype Elgersma, J. Gerard G. Borst

**Affiliations:** ^1^Department of Neuroscience, Erasmus MC, University Medical Center RotterdamRotterdam, Netherlands; ^2^Department of Clinical Genetics, Erasmus MC, University Medical Center RotterdamRotterdam, Netherlands; ^3^ENCORE Expertise Center for Neurodevelopmental disorders, Erasmus MC, University Medical Center RotterdamRotterdam, Netherlands

**Keywords:** mTOR signaling cascade, autism spectrum disorders, calyx of Held, synaptic transmission, synaptic morphology, intellectual disability, juxtacellular recording, short-term plasticity

## Abstract

Defects in the rat sarcoma viral oncogene homolog (Ras)/extracellular-signal-regulated kinase and the phosphatidylinositol 3-kinase-mammalian target of rapamycin (mTOR) signaling pathways are responsible for several neurodevelopmental disorders. These disorders are an important cause for intellectual disability; additional manifestations include autism spectrum disorder, seizures, and brain malformations. Changes in synaptic function are thought to underlie the neurological conditions associated with these syndromes. We therefore studied morphology and *in vivo* synaptic transmission of the calyx of Held synapse, a relay synapse in the medial nucleus of the trapezoid body (MNTB) of the auditory brainstem, in mouse models of tuberous sclerosis complex (TSC), Fragile X syndrome (FXS), Neurofibromatosis type 1 (NF1), and Costello syndrome. Calyces from both *Tsc1^+/-^* and from *Fmr1* knock-out (KO) mice showed increased volume and surface area compared to wild-type (WT) controls. In addition, in *Fmr1* KO animals a larger fraction of calyces showed complex morphology. In MNTB principal neurons of *Nf1*^+/^*^-^* mice the average delay between EPSPs and APs was slightly smaller compared to WT controls, which could indicate an increased excitability. Otherwise, no obvious changes in synaptic transmission, or short-term plasticity were observed during juxtacellular recordings in any of the four lines. Our results in these four mutants thus indicate that abnormalities of mTOR or Ras signaling do not necessarily result in changes in *in vivo* synaptic transmission.

## Introduction

Neurodevelopmental disorders with clinical overlap often share defects within the same intracellular signal transduction pathway. A lot of progress has recently been made in the identification of mutations affecting the rat sarcoma viral oncogene homolog (Ras)/extracellular-signal-regulated kinase (ERK) and the phosphatidylinositol 3-kinase (PI3K)-mammalian target of rapamycin (mTOR) signaling pathways, which are responsible for several neurodevelopmental disorders (Krab et al., 2008). Here, we studied *in vivo* synaptic transmission in mouse models for four different monogenetic syndromes in which these two pathways are affected: tuberous sclerosis complex (TSC), Fragile X syndrome (FXS), Neurofibromatosis type 1 (NF1), and Costello syndrome (CS). In TSC and FXS the mTOR pathway is upregulated (Ebrahimi-Fakhari and Sahin, 2015). The mTOR pathway plays a central role in synaptic plasticity, and its upregulation is linked to intellectual disability, autism spectrum disorder, seizures, and brain malformations (Swiech et al., 2008; Crino, 2011; Troca-Marín et al., 2012; Lasarge and Danzer, 2014). In NF1, FXS, and CS the Ras/ERK pathway is upregulated as well (Osterweil et al., 2013; Rauen, 2013). At the synaptic level, these four syndromes are characterized by abnormal synaptic plasticity and often abnormal synaptic morphology (Swiech et al., 2008; Stornetta and Zhu, 2011; Levenga and Willemsen, 2012; Rauen, 2013).

Tuberous sclerosis complex is the most well-known syndrome linked to upregulation of the mTOR pathway (Crino, 2011). TSC is characterized by the formation of benign tumors in several organs, as well as brain malformations and neuronal defects (Crino, 2011; Lasarge and Danzer, 2014). The most important neurological manifestations are seizures, intellectual disability, autism, and hydrocephalus (Krab et al., 2008; Crino, 2011; Troca-Marín et al., 2012; Lasarge and Danzer, 2014; Ebrahimi-Fakhari and Sahin, 2015). In addition, deficits in cortical auditory evoked potentials have been observed in TSC patients with autistic behavior (Seri et al., 1999). TSC results from spontaneous or inherited mutations in *TSC1* or *TSC2*. The proteins encoded by the *TSC1* and *TSC2* genes, hamartin, and tuberin, respectively, form a complex that inhibits ras homologue expressed in brain (RHEB), an activator of the mTOR. Loss of function mutations in *TSC1* or *TSC2* thus lead to disinhibition of mTOR (Kassai et al., 2014; Ma et al., 2014). At the synaptic level, this disinhibition leads to increased axonal collateral formation, extended projections, and more and larger synapses in *Drosophila* (Canal et al., 1998; Acebes and Ferrús, 2001; Knox et al., 2007). Whether or not this leads to increases in synaptic transmission depends on the exact mutation and the downstream pathway that is altered. Presynaptic overexpression of RHEB leads to enhanced synaptic function, whereas *TSC2* null mutants do not show increased quantal output at the *Drosophila* neuromuscular junction (Knox et al., 2007; Natarajan et al., 2013). In agreement with the results in flies, the effects of mutations in *Tsc1* or *Tsc2* on excitatory transmission are typically modest or absent in rodents. No net change in glutamatergic synapse-driven excitability was observed in isolated *Tsc1* KO neurons, autaptic hippocampal neurons, or Purkinje cells (Tsai et al., 2012; Bateup et al., 2013; Weston et al., 2014). Normal basal synaptic transmission was observed in the hippocampus of *Tsc2*^+/-^ mice and upon acute deletion of *Tsc1* (Ehninger et al., 2008; Abs et al., 2013). In contrast to the absence of obvious changes in basal excitatory synaptic transmission, clear changes in long-term plasticity have been observed in *Tsc2^+/^*^-^ Eker rats (von der Brelie et al., 2006), *Tsc2*^+/-^ mice (Ehninger et al., 2008), and in the absence of *Tsc1* in mice (Bateup et al., 2011; Abs et al., 2013).

Fragile X syndrome is caused by loss of function of the fragile X mental retardation protein (FMRP; Pieretti et al., 1991; Verkerk et al., 1991), which acts as a translational repressor of specific mRNAs (Corbin et al., 1997; Feng et al., 1997; Ascano et al., 2012). The absence of FMRP leads to an upregulation of many proteins, including PI3K and mTOR (Gross et al., 2010; Sharma et al., 2010; Ascano et al., 2012; Bhakar et al., 2012). FXS is the leading cause of inherited intellectual disability; other neurologic manifestations include autism, anxiety, and ADHD (Boyle and Kaufmann, 2010). In addition, individuals with FXS frequently have sensory processing and sensory integration problems (Miller et al., 1999; Frankland et al., 2004). Auditory processing deficits, including auditory hypersensitivity and reduced habituation, are a prominent feature of FXS (Rotschafer and Razak, 2014). Patients with FXS have abnormalities in the morphology of dendritic spines and in a mouse model for the disease there are more immature spines in hippocampus and neocortex, and the total number of dendritic spines is increased (Levenga and Willemsen, 2012). A large variety of changes in synaptic transmission have been observed in the mouse model for FXS, including changes in short- and in long-term plasticity (Bhakar et al., 2012). Most of them are thought to be secondary to the effects of FMRP on translation, but some of them are caused by a direct binding of FMRP to proteins involved in synaptic transmission (Brager and Johnston, 2014).

Neurofibromatosis type 1 is caused by heterozygous mutations in the *Nf1* gene. *Nf1* encodes the protein neurofibromin, which functions as a Ras-GTPase activating protein (DeClue et al., 1991; Gutmann et al., 1991). *Nf1* mutations lead to abnormally high Ras activity, which in its turn leads to increased PI3K and ERK 1/2 activity (Basu et al., 1992; DeClue et al., 1992). These latter two proteins have many different functions including the inactivation of the TSC1/TSC2 complex, thus releasing inhibition of RHEB and allowing activation of mTOR (Krab et al., 2008). The most commonly observed neurological abnormalities in NF1 are behavioral abnormalities and mild intellectual disability (Rauen, 2013). Auditory temporal processing deficits have been observed in NF1 patients (Batista et al., 2014). Mice with heterozygous KO of *Nf1* (*Nf1^+/-^*) can serve as an animal model for NF1 (Jacks et al., 1994; Silva et al., 1997; Shilyansky et al., 2010b). Many of the neurological symptoms of these mice are caused by increased synaptic inhibition leading to impairments in long-term potentiation (LTP; Costa et al., 2002; Cui et al., 2008; Shilyansky et al., 2010a; Molosh et al., 2014; Omrani et al., 2015). To what extent the changes in synaptic transmission are accompanied by morphological changes is unclear, but cultured neurons from *Nf1*^+/^*^-^* mice have reduced neurite length and smaller growth cones (Brown et al., 2010a).

Costello syndrome is caused by heterozygous germ line gain of function mutations in the proto-oncogene *H-ras* (Aoki et al., 2005). Its neurological phenotype includes mild to moderate intellectual disability and increased anxiety (Axelrad et al., 2011). Infants with CS generally show characteristic irritability, which includes hypersensitivity to sound (Kawame et al., 2003). Transgenic mouse models postnatally expressing constitutively active H-ras with a Gly12Val mutation (*H-ras^G12V^*) show increased vesicle docking, changes in short-term plasticity and increased LTP (Seeger et al., 2004; Kushner et al., 2005). A knock-in mouse with the same mutation mimics many of the neurological deficits associated with Costello syndrome (Viosca et al., 2009). No abnormalities in acoustic startle reflex or prepulse inhibition of the startle reflex were observed (Viosca et al., 2009). Little is known about the changes in synaptic morphology or transmission in these mice. Therefore, we also included these mice in our study.

The proteins that are responsible for TSC, FXS, NF1, and CS (hamartin or tuberin, FMRP, neurofibromin and H-ras) are ubiquitously expressed in neurons (Furth et al., 1987; Daston and Ratner, 1992; Bakker et al., 2000; Gutmann et al., 2000). Most of the characterizations of functional synaptic abnormalities have been performed in slices or *in vitro* systems using cultured primary neurons from mutant mice. The principal aim of this work was to understand how these neurogenetic disorders affect firing behavior and synaptic transmission *in vivo*. To achieve this goal, we used the calyx of Held synapse as a model synapse. The calyx of Held synapse is a giant, glutamatergic synapse formed between a globular bushy cell of the anteroventral cochlear nucleus (AVCN) and a principal neuron of the medial nucleus of the trapezoid body (MNTB). It functions as a relay synapse in the auditory brainstem; the glycinergic principal neurons of the MNTB provide inhibition that is both well timed and sustained to many other auditory nuclei (Borst and Soria van Hoeve, 2012). We focus on *in vivo* transmission at the calyx of Held synapse for two reasons. Firstly, intellectual disability is associated with a relatively high prevalence of sensory abnormalities (Carvill, 2001). For instance, *Fmr1* KO mice have an enhanced auditory startle response and prepulse inhibition and increased auditory seizures (Rotschafer and Razak, 2014). Secondly, because of its giant size and accessibility, the calyx of Held synapse can be used in *in vivo* juxtacellular recordings for quantitative studies of synaptic transmission, making it highly suitable for *in vivo* screening for deficits in synaptic transmission (Lorteije et al., 2009). A slice study has shown a correlation between the complexity of the calyceal morphology and its function (Grande and Wang, 2011). Calyces with relatively simple morphologies exhibit comparatively large short-term synaptic depression (STD) and a high percentage of spike failures, whereas structurally complex calyces are associated with facilitation, followed by slow depression and almost no spike failures. We therefore also compared the morphology of the calyx in WT and mutant mice in these four lines.

In the present study, we found that calyces from *Tsc1^+/-^* and *Fmr1* KO mice had larger volume and surface area, and that the calyces of *Fmr1* KO mice showed more boutons compared to WT littermates. Calyx morphology of the *Nf1^+/-^* and *H-ras^G12V^* mutant mice was similar to their WT littermates. In addition, the *in vivo* juxtacellular recordings revealed that the delay between EPSPs and APs was slightly smaller in recordings from the *Nf1^+/-^* mice.

## Materials and Methods

### Animals and Experiments

Generation of the *Fmr1* KO (Mientjes et al., 2006), *Nf1^+/-^* (Jacks et al., 1994), and *H-ras^G12V^* mice (Schuhmacher et al., 2008) has been described previously. *Tsc1^+/-^* animals were obtained by crossing the *Tsc1^f/+^* line (Meikle et al., 2005) once with CAGG-Cre mice to obtain germ-line deletion. Animals were all backcrossed (for at least 10 times) and maintained in C57BL/6J (Harlan). Wild-type (WT) littermates were used as controls. Mutant and WT animals of either sex were used for the experiments. All experiments were conducted in accordance with the European Communities Council Directive and were approved by the Animal Ethics Committee of the Erasmus MC.

### Surgery

Adult mice (30 to 60 days-old) were briefly exposed to isoflurane before they were anesthetized by giving an intraperitoneal injection of a ketamine–xylazine mixture (65 and 10 mg/kg, respectively). Additional anesthesia was given when necessary. A homeothermic blanket (FHC, Bowdoinham, ME, USA) was used to keep the rectal temperature at 36–37°C. Animals were supine positioned, and a tracheotomy was performed so that the animal could be ventilated mechanically with oxygen (MiniVent; type 845; Harvard Apparatus). A small speaker was glued to the inside of the left ear if sound exposure was intended. Animals got a metal pedestal glued to the dorsal side of the skull, which was used to immobilize the head when they were positioned supinely. After doing a tracheotomy, the mice were mechanically ventilated with oxygen. Ventilation frequency and breathing volume were set to approximately 100 strokes/min and 7 μl/g body weight, respectively. The MNTB was accessed ventrally as described previously (Rodriguez-Contreras et al., 2008; Lorteije et al., 2009; Crins et al., 2011). In short, the trachea, larynx, and muscles covering the skull were removed, after which a small craniotomy (1 mm × 1.5 mm) was made by using a drill. The right anterior inferior cerebellar artery (AICA) and basilar artery were used as landmarks to locate the right MNTB, as previously described (Rodriguez-Contreras et al., 2008; Crins et al., 2011). Prior to recording, the dura and pia were removed to expose the brain surface. Ringer solution containing (in mM): NaCl 135, KCl 5.4, MgCl_2_ 1, CaCl_2_ 1.8, Hepes 5 (pH 7.2 with NaOH) was applied to keep the brain surface moist.

### *In Vivo* Juxtacellular Recordings

*In vivo* juxtacellular (loose patch) recordings of the principal neurons were performed as described previously (Lorteije et al., 2009). During recordings, Ringer solution was applied onto the brain surface to prevent it from dehydration. Recordings were done using thick-walled borosilicate glass micropipettes with filament (4.5–5.5 MΩ) filled with Ringer solution. The MNTB was accessed ∼600 μm rostrally and ∼400 μm laterally from the branching point of the right AICA from the basilar artery. The dura and brain surface were penetrated using a positive pressure of 300 mbar. When inside the brainstem the pressure was lowered to ∼30 mbar after passing the brain surface, and to 0 mbar when recording started. Recordings were performed at the depth of 250–500 μm from brain surface. A MultiClamp 700B patch-clamp amplifier and pCLAMP 9.2 software (MDS Analytical Technologies) were used to acquire data. Data was filtered at 10 kHz (8-pole Bessel filter) and sampled at 50 kHz with a 16-bit A/D converter (Digidata 1322A).

### Auditory Stimulation

Closed field sound stimulation was presented to the animal, as described previously (Tan and Borst, 2007). During the experiments the animal was placed in a single-walled sound-attenuated chamber (Gretch-Ken Industries). A speaker probe was inserted into the left ear canal and was stabilized with silicone elastomer. A 2-noise-burst stimulation protocol was designed in MATLAB (Version R2008a), which consists of two 400 ms bursts of wide band noise (bandwidth 2–40 kHz; 80 dB SPL) with intervals of 40, 80, 160, 320, 640, or 1280 ms. The auditory stimulus was generated by Tucker Davis Technologies hardware (TDT, system 3, RX6 processor, PA5.1 attenuator, ED1 electrostatic driver, EC1 electrostatic speaker). Clampex acquisition was triggered at the same time by MATLAB as the auditory stimulation program. Sound intensities were calibrated as previously described (Tan and Borst, 2007).

### Morphology of the Calyx

To study the morphology of the calyx, we electroporated the afferent fibers to the calyx of Held with Alexa Fluor 594-labeled dextrans (10,000 MW; Invitrogen) at the midline *in vivo* in young adult mice (P28–P84) as described previously (Rodriguez-Contreras et al., 2008). The ages of the animal were not exactly matched for the *Tsc1^+/-^* and the *H-ras^G12V^* mice and their respective WT controls (Supplementary Figure S1). However, these age differences are unlikely to be meaningful, since we did not see a relation between volume of the calyx with age (*r* = 0.032; *p* = 0.73; Supplementary Figure S2) or between surface and age (*r* = 0.00; *p* = 0.99; Supplementary Figure S3). In addition, logistic regression showed no evidence for a relation between probability of being classified as type II (6–15 boutons; (Grande and Wang, 2011) or type III (>15 boutons) and age (*p* = 0.46; Supplementary Figure S4).

The animal was perfused and the brainstem was sliced into 40-μm-thick sections 1 h after electroporation. We applied the same fixation time for all the slices, and no significant shrinkage was observed. To acquire high resolution *z*-stack images of the calyces, a laser scanning confocal microscope (LSM 700; Zeiss) equipped with krypton-argon and helium-neon lasers was used (0.5 μm steps; 63x oil immersion objective, NA 1.4). Optimized laser power, detector gain, and pinhole diameter settings were applied during image acquisition. Calyces with a signal-to-noise ratio of >15 were randomly selected (Di Guilmi et al., 2014). The number of swellings per calyx was counted on 3D-rendered (Volocity 4.2; Improvision) images of calyces with adjusted contrast and brightness. The surface area and volume of the calyces were measured using the region-of-interest function in Volocity; images of calyx terminals were binary thresholded using the built-in thresholding function of ImageJ 1.46 (isodata algorithm). As a measure for the size of principal neurons, we identified the slice with the largest cross-section in image stacks and calculated the neuron’s area assuming an elliptic shape (Sommer et al., 1993) using the formula: area = π^∗^a^∗^b, in which a is the length of the semi-major axis and b the length of the semi-minor axis.

### Analysis

Electrophysiological data was analyzed with a custom written program in Igor 6.22A (WaveMetrics) NeuroMatic (version 2.00, kindly provided by Dr. J. Rothman, University College London, London, UK). The genotypes were revealed after completion of analysis. All *in vivo* recordings reported in this paper showed evidence for the presence of a prespike (Guinan and Li, 1990). Complex extracellular waveforms were analyzed as described previously (Lorteije et al., 2009). The first, smaller, positive deflection, which is called the prespike (Forsythe, 1994), originates from the calyx of Held (Lorteije et al., 2009). Its peak coincides with the maximum rate of rise of the presynaptic action potential (Borst et al., 1995). The second, larger, positive deflection originates from the principal neuron. It typically consists of two parts, an early part, which is the extracellularly recorded EPSP (eEPSP) and, if the EPSP is suprathreshold, the extracellularly recorded AP (eAP; Lorteije et al., 2009). Juxtacellular recordings measure local membrane currents, which consist of capacitive and resistive currents (Freygang and Frank, 1959). We showed previously that the amplitude of the first component provides a good (relative) measure for the rate of rise of the intracellular EPSP, and that the same holds for its derivative (eEPSP’) and that both also provide a measure for transmitter release (Lorteije et al., 2009), since postsynaptic saturation and desensitization are not an issue in the adult calyx of Held (Yamashita et al., 2003; Renden et al., 2005). Similarly, the peak of the eAP, which typically coincides with the maximum rate of rise of the postsynaptic AP, provides a measure for the postsynaptic excitability. Because of the difficulty of delineating the amplitude of the eEPSP from the eAP, we used the eEPSP’ as a measure for the strength of transmission, as previously described (Wang et al., 2013). The absolute size of the eEPSP’ depends on experimental parameters such as seal resistance; on average it was about 10 V/s; this value did not differ significantly between mutant and its WT control for any of the four lines (Supplementary Figure S5). The half width of the eAP was measured halfway between the local baseline and the peak of the eAP. It provides a measure for the duration of the fast depolarization phase. The eEPSP-eAP delay is the delay between the eEPSP’ and the eAP; it thus provides a measure for the ease at which an EPSP triggers an AP. Synaptic delay was defined as the latency between the peak of the prespike and eEPSP onset; eEPSP onset was defined as the point in time at which a line through max rate of rise point and the point at which rate of rise was half-maximal crosses the baseline level. Prespike-eAP delay, eEPSP-AP delay, and half width of the eAP were measured in spontaneous events.

Event amplitudes were fitted with a simple model for STP (Varela et al., 1997). In the simplest form of this model, a single depression state parameter decreases at each event with a fraction called the depletion factor (comparable to the release probability of vesicles in the readily releasable pool) and recovers continuously with a single time constant. Synaptic transmission is equal to the product of the depression state parameter and the transmission strength in the absence of STP.

Data is presented as the mean ± SEM. Statistical significance of differences between means was assessed using ANOVA for the morphological differences and multivariate analysis of variance (MANOVA), followed by (univariate) ANOVA and Student’s *t*-test for the electrophysiological differences. In order to test if there was any significant finding in our data set of all four mutant groups, the MANOVA tested whether the mutants differed from their controls with respect to the measured parameters eEPSP-eAP delay, half width of eAP, spontaneous frequency, maximum evoked frequency, steady-state frequency, facilitation, amount of STD, while controlling for background strain effects. The variance–covariance matrices were not homogenous and hence Pillai’s trace was utilized to determine significance of the MANOVA. The prespike-eAP delay, time constant of depression and time constant of recovery from STD were not included in the MANOVA because these values could not be measured in many cells. Spontaneous failure percentage was also not included, since its distribution was highly skewed.

## Results

### Calyx of Held Morphology is altered in *Tsc1^+/-^* and *Fmr1* KO Mice

Syndromes associated with intellectual disability often show abnormal synaptic morphology (Levenga and Willemsen, 2012). To address if the morphology of the calyx of Held synapse is altered in mouse models for TSC, FXS, NF1, or CS, we electroporated axonal fibers *in vivo* at the midline with Alexa Fluor 594-labeled dextrans in young-adult animals (P28–P84). We then made image stacks of labeled calyces under the confocal microscope. Volume and surface area were measured and the number of boutons was counted as a measure for the morphological complexity of the calyx. Calyces were divided in three groups containing either <6 (type I), 6–15 (type II) or >15 (type III) boutons (Grande and Wang, 2011). However, we did not observe type I calyces in any of the four lines in either WT or mutant mice (*n* = 107).

**FIGURE 1:**
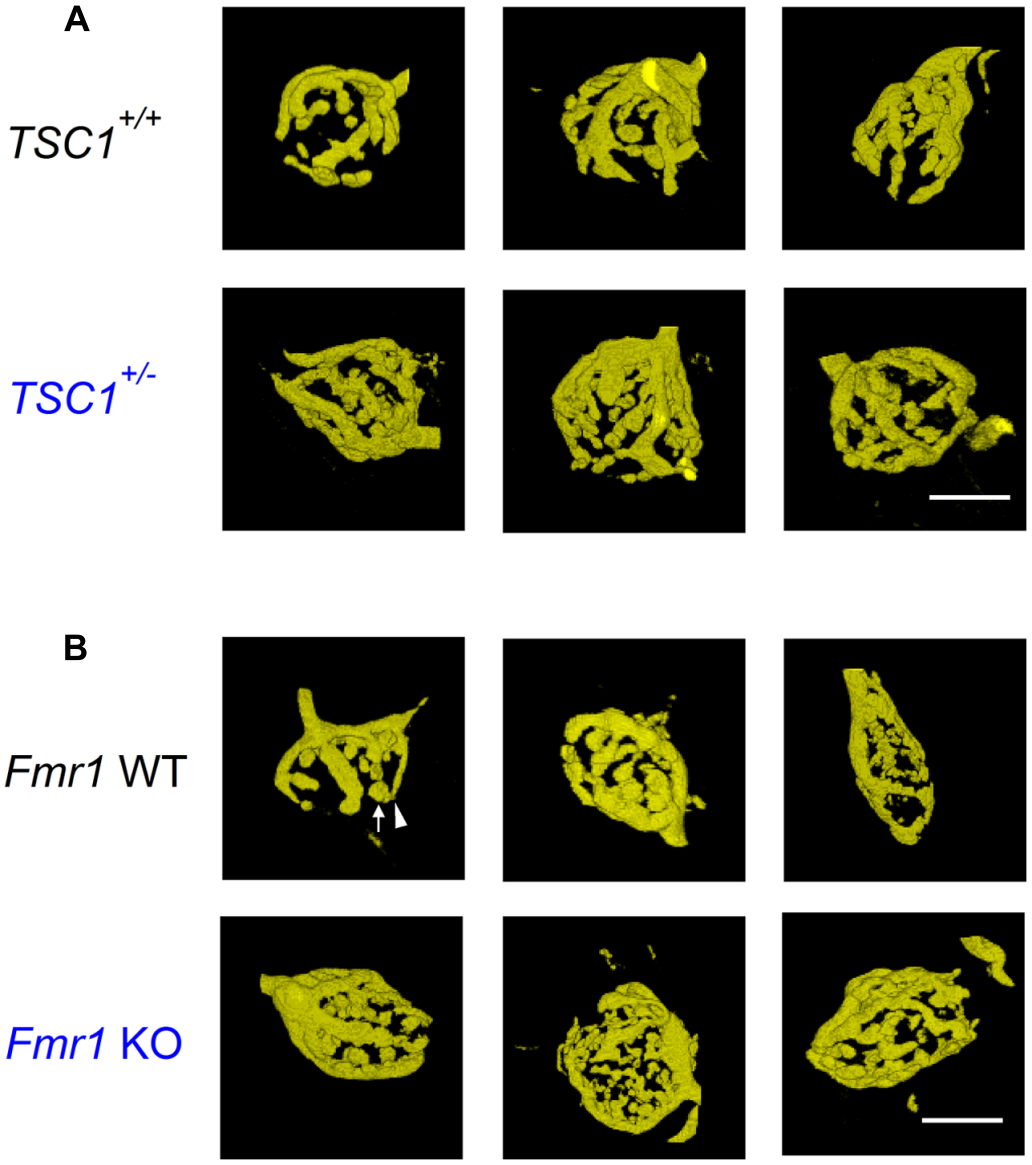
**Calyx of Held morphology of *Tsc1^+/-^* and *Fmr1* KO mutant mice. (A)** Example images of representative, 3D-rendered calyces from wild-type (WT; top row) and *Tsc1^+/-^* (bottom row) mice. **(B)** Images from WT (top row) and *Fmr1* KO mutant mice (bottom row). Arrow indicates bouton; arrowhead neck. Scale bars 10 μm.

**FIGURE 2:**
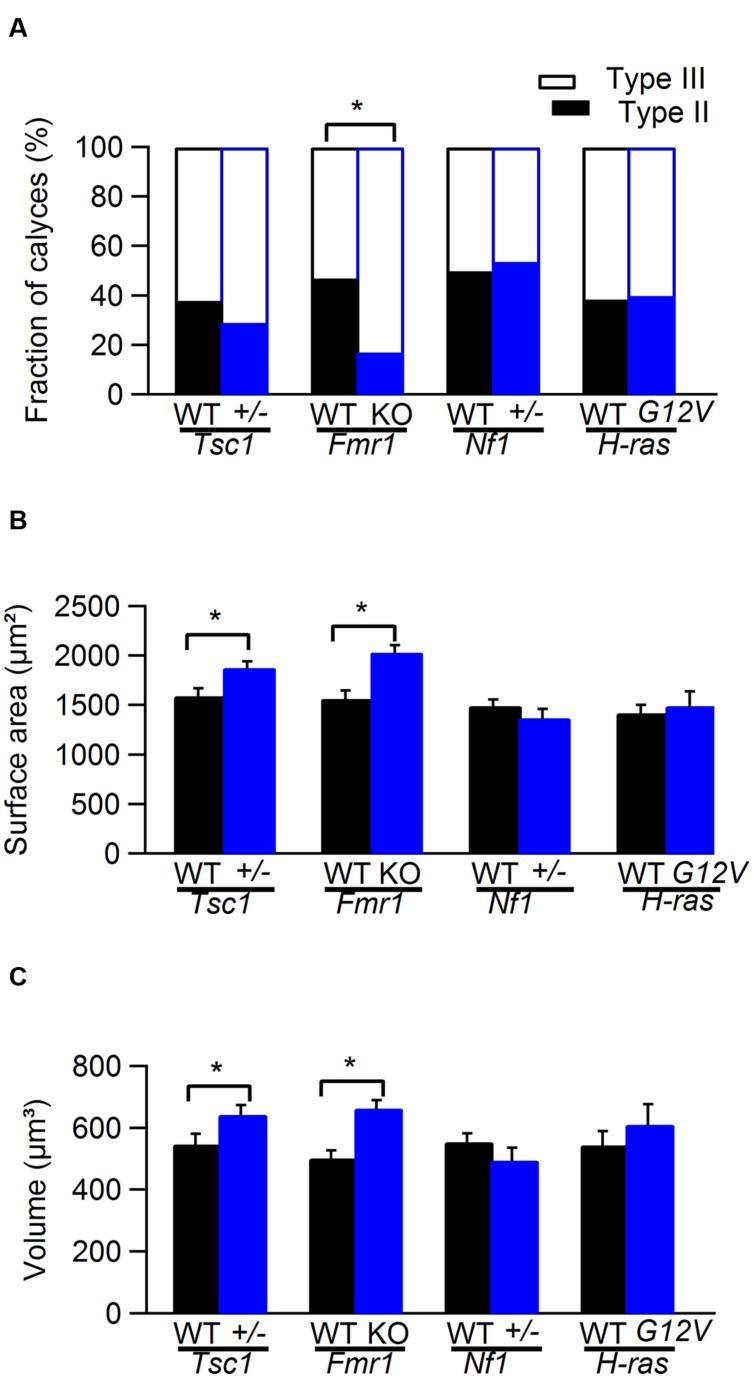
**Comparison of calyx of Held morphology from WT and mutant mice. (A)** Comparison of the fraction of type II and type III calyces in WT (black) and *Tsc1^+/-^*, *Fmr1* KO, *Nf1^+/-^*, and *H-ras^G12V^* mutant mice (blue). **(B)** Comparison of the surface area of calyx of Held synapse in WT (black) and *Tsc1^+/-^*, *Fmr1* KO, *Nf1^+/-^*, and *H-ras^G12V^* mutant mice (blue). **(C)** Comparison of the volume of calyx of Held synapse in WT (black) and *Tsc1^+/-^*, *Fmr1* KO, *Nf1^+/-^*, and *H-ras^G12V^* mutant mice (blue). ^∗^ indicates significant difference. Error bars indicate SEM.

Representative examples of calyces in *Tsc1^+/-^* and in *Fmr1* KO mice and their respective controls are shown in **Figure 1**. In *Tsc1^+/-^* mice, calyces had both a larger volume and an increased surface area (548 ± 34 μm^3^ in WT, *n* = 14 vs. 643 ± 31 μm^3^ in *Tsc1^+/-^*, *n* = 13; *p* = 0.05; 1592 ± 76 μm^2^ in WT vs. 1880 ± 62 μm^2^ in *Tsc1^+/-^*; *p* = 0.01; **Figures 1A and 2B,C**). This increase was unlikely to be due to a general increase in cell size, since maximum postsynaptic surface cell area was not different in WT (331 ± 12 μm^2^; *n* = 30) compared to *Tsc1^+/-^* (355 ± 11 μm^2^; *n* = 27; *p* = 0.16). The fraction of type III calyces was similar in *Tsc1^+/-^* mice and their WT controls (**Figure 2A**). Calyces from *Fmr1* KO mice also showed both larger volume and surface area (502 ± 25 μm^3^ in WT, *n* = 19 vs. 663 ± 26 μm^3^ in *Fmr1* KO, *n* = 29; *p* = 0.013; 1564 ± 79 μm^2^ in WT vs. 2032 ± 71 μm^2^ in *Fmr1* KO; *p* = 0.036; **Figures 1B and 2B,C**). Similar to the *Tsc1^+/-^* mice, no change in maximum postsynaptic cross-section area was observed in WT (320 ± 11 μm^2^; *n* = 43) compared to the *Fmr1* KO (314 ± 12 μm^2^; *n* = 38; *p* = 0.70). In *Fmr1* KO animals, a larger fraction of calyces were type III than in control (83% vs. 53%; *p* = 0.025; **Figure 2A**).

No obvious morphological differences were observed between *Nf1^+/-^* mice and their WT controls or between and *H-ras^G12V^* mice and their WT controls (**Figure 2**).

### Action Potential Waveforms and Firing Behavior

**FIGURE 3:**
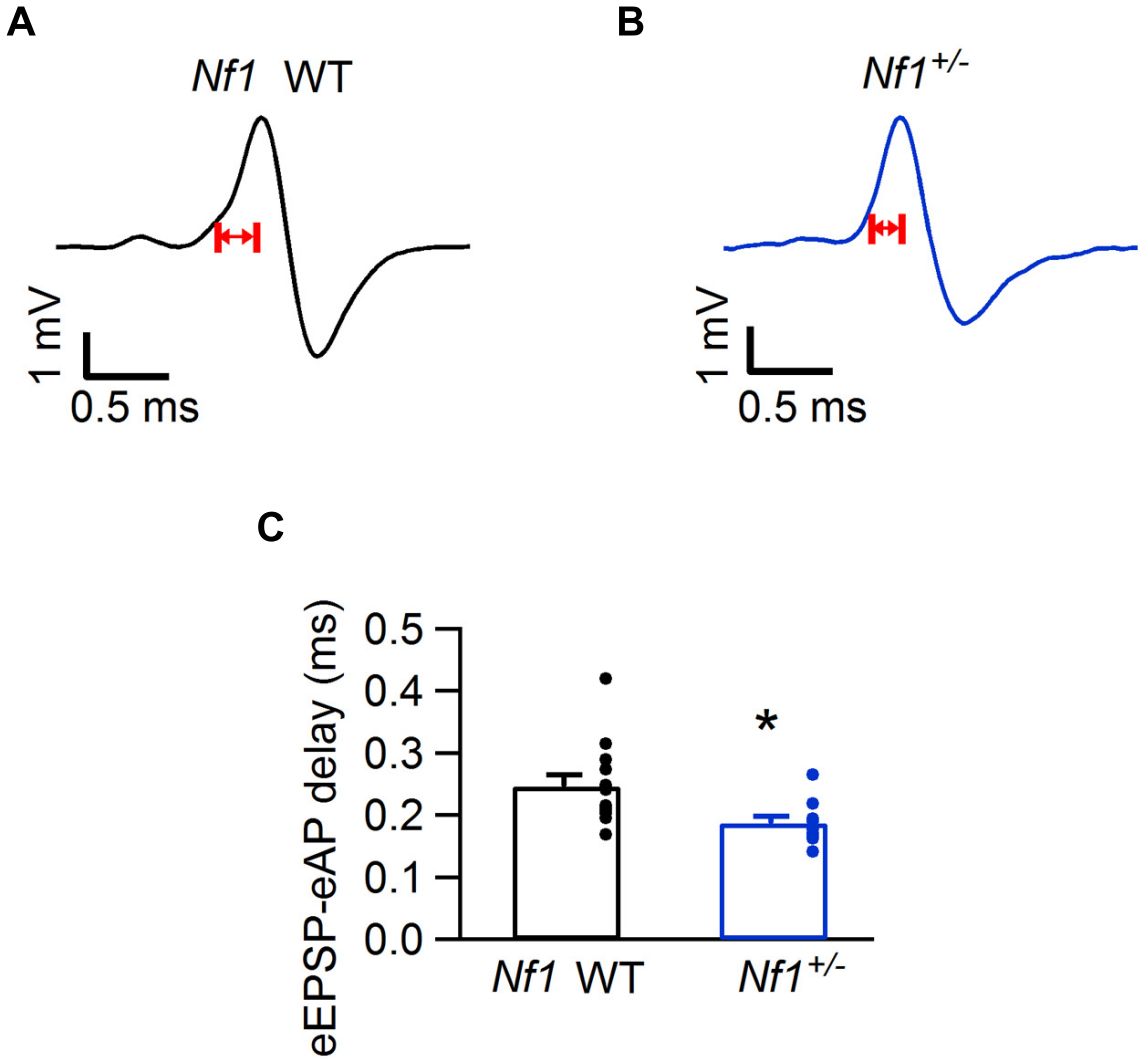
**Decreased eEPSP-eAP delay in *Nf1^+/-^* mice. (A)** Representative juxtacellular complex waveform from a *Nf1^+/+^* (wild-type) mouse. Red arrows indicate the eEPSP-eAP delay. **(B)** Same as **(A)**, but from a *Nf1^+/-^* mouse. **(C)** Comparison of eEPSP-eAP delay of medial nucleus of the trapezoid body (MNTB) neurons between *Nf1^+/+^* wild-type (black) and mutants (blue). Significant difference is indicated with ^∗^. Error bars indicate SEM; individual data points are shown as small filled circles.

We next studied the *in vivo* firing behavior of the calyx of Held synapse. To that end, we made juxtacellular recordings from the MNTB of anesthetized mutant and littermate WT animals. Recordings from the calyx of Held synapse are characterized by the presence of a complex extracellular waveform (Guinan and Li, 1990), which consists of two positive brief deflections originating from the calyx of Held and the principal cell, respectively (Lorteije et al., 2009). An example is shown in **Figure 3A**. Waveform analysis yielded eEPSP-eAP delay and eAP half width. In cells in which the size of the prespike was sufficiently large, prespike-eAP delay was also quantified. We also measured spontaneous firing frequency and the steady state firing frequency during presentation of a 400 ms, 80 dB noise burst. Finally, we measured the fraction of subthreshold eEPSPs during spontaneous activity. The results of the analysis of the shape of the complex extracellular waveforms and the firing behavior are summarized in **Table 1**.

**Table 1:** Analysis of complex waveforms and STP in four different mouse models for neuropsychiatric disorders.

	Prespike-eAP delay (ms)	eEPSP-eAP delay (ms)	eAP half width (ms)	Spontaneous frequency (Hz)	Maximum evoked frequency (Hz)	Steady state firing frequency (Hz)	Spontaneous failures (%)	Facilitation	STD (Relative eEPSP’)	Depression τ (number of events)	Recovery τ (ms)
*Tsc1^+/+^*	0.55 ± 0.02	0.2 ± 0.01	0.26 ± 0.01	21 ± 3	245 ± 32	131 ± 18	0.01 ± 0.01	1.06 ± 0.01	0.84 ± 0.02	6 ± 0.5	207 ± 74
*Tsc1^+/^^-^*	0.68 ± 0.04	0.25 ± 0.03	0.26 ± 0.02	30 ± 9	288 ± 32	148 ± 19	8.28 ± 6.32	1.07 ± 0.03	0.78 ± 0.05	6.6 ± 1.4	274 ± 91
*Fmr1* WT	0.65 ± 0.02	0.25 ± 0.02	0.27 ± 0.01	23 ± 5	303 ± 22	159 ± 9	2.31 ± 2.28	1.07 ± 0.01	0.81 ± 0.03	6.4 ± 0.7	177 ± 43
*Fmr1* KO	0.65 ± 0.03	0.23 ± 0.01	0.26 ± 0.01	27 ± 6	282 ± 24	145 ± 12	0.17 ± 0.12	1.08 ± 0.02	0.76 ± 0.03	6.6 ± 0.6	245 ± 96
*Nf1^+/+^*	0.72 ± 0.04	0.25 ± 0.02	0.25 ± 0.01	16 ± 5	326 ± 11	151 ± 6	8.12 ± 5.77	1.15 ± 0.04	0.67 ± 0.04	4.9 ± 0.3	227 ± 46
*Nf1^+/^^-^*	0.63 ± 0.03	0.18 ± 0.01^∗^	0.22 ± 0.01	37 ± 12	305 ± 17	162 ± 12	0.85 ± 0.85	1.14 ± 0.03	0.75 ± 0.04	5 ± 0.6	219 ± 60
*Hras* WT	0.72 ± 0.04	0.23 ± 0.02	0.26 ± 0.02	40 ± 7	302 ± 17	155 ± 12	1.68 ± 1.47	1.1 ± 0.03	0.78 ± 0.04	4.9 ± 0.4	156 ± 74
*Hras^G12V^*	0.76 ± 0.05	0.24 ± 0.02	0.22 ± 0.01	36 ± 10	261 ± 14	135 ± 12	3.86 ± 2.21	1.13 ± 0.05	0.82 ± 0.03	6.2 ± 0.8	155 ± 74

Different parameters were quantified in in vivo recordings from Tsc1^+/-^, Fmr1 KO, Nf1^+/-^, and H-ras^G12V^ mutant mice and their respective wild-type (WT) littermates (n = 12 for Tsc1 WT and n = 9 for Tsc1^+/-^; n = 26 for Fmr1 WT and n = 18 for Fmr1 KO; n = 13 for Nf1 WT and n = 10 for Nf1^+/-^; n = 10 for H-ras WT and n = 9 for H-ras^G12V^). Data is presented as mean ± SEM. * indicates significant difference in pair-wise post hoc t-test comparison.

The electrophysiological properties of mutants differed significantly from their wild-type controls (MANOVA; *p* = 0.027; Pillai’s trace). This difference could be largely attributed to the eEPSP-eAP delay (ANOVA; *F*_4,99_ = 3.3, *p* = 0.015). Individual *t*-tests on the eEPSP-eAP delays in the four mutant lines indicated that this delay was significantly smaller in the *Nf1*^+/-^ mice compared to their WT controls (**Figure 3**; **Table 1**; *p* = 0.036; *p*-value Bonferroni-corrected for multiple testing).

The shorter eEPSP-eAP delay suggests a gain-of-function phenotype in the *Nf1*^+/-^ mice. The average failure rate indeed was somewhat lower in the *Nf1*^+/-^ mice than in their WT controls (0.85 ± 0.85% vs. 8.1 ± 5.8%; *p* = 0.41; Mann–Whitney *U* test), and in the *Nf1*^+/-^ mice only two out of ten cells showed failures, whereas five out of thirteen cells showed failures in the WT controls during spontaneous activity.

**FIGURE 4:**
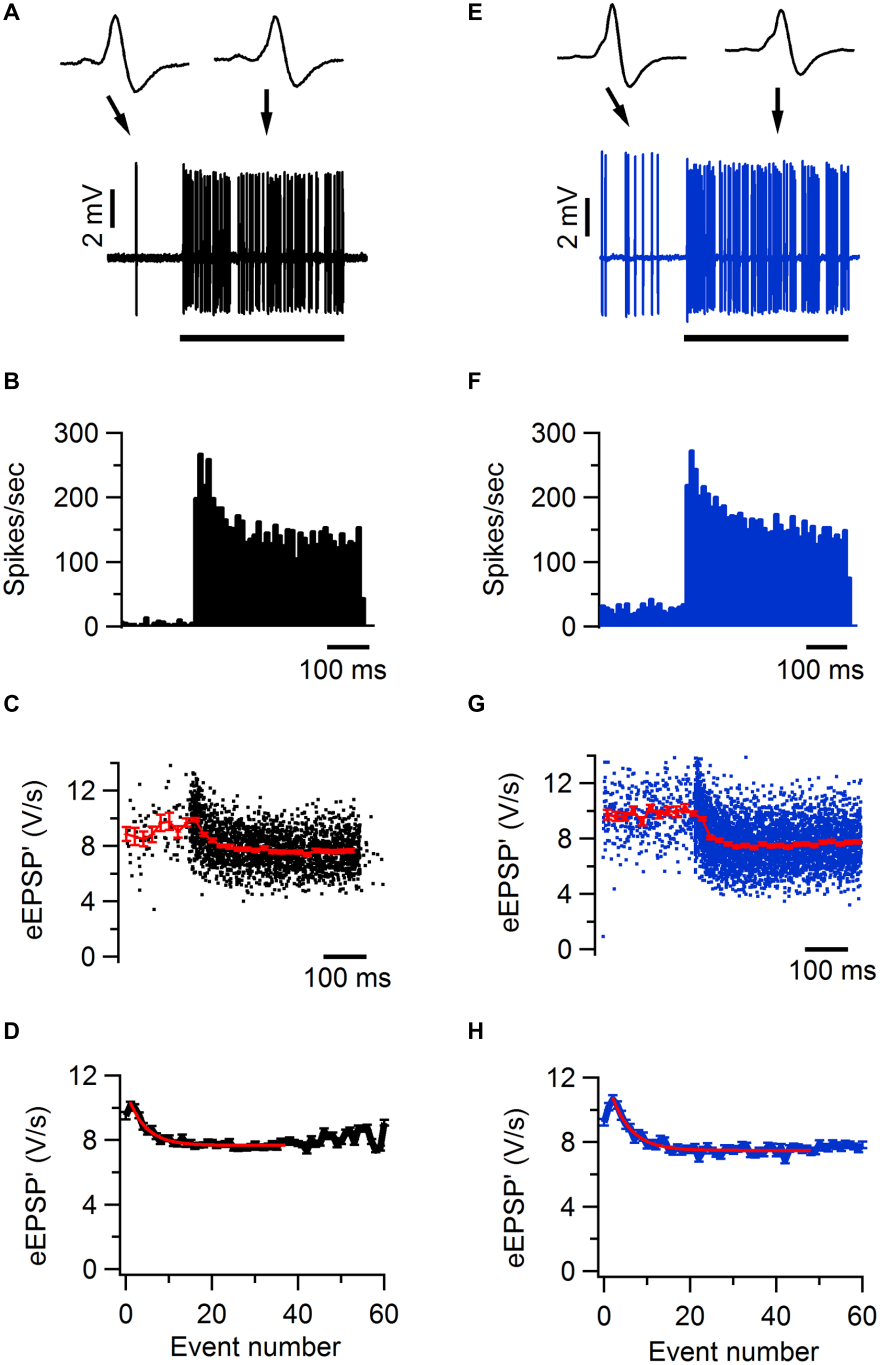
**Sound-evoked STD in *Tsc1^+/-^* and wild-type mouse. (A)** Increase in firing frequency during a 400 ms, 80 dB noise burst in a juxtacellular recording from a wild-type mouse. Insets show two juxtacellular complex waveforms before and during sound stimulation, respectively. Black bar indicates the presentation of the sound stimulus. **(B)** Peristimulus time histogram showing primary-like response to sound. **(C)** Maximal amplitude of the first derivative of the eEPSP (eEPSP’; black) and binned average ± SEM (red). **(D)** Average amplitude of eEPSP’ against sound-evoked event number, where event 0 is the average amplitude during the baseline period and event 1 is the second event evoked after sound onset. Red line indicates the fit with a single exponential function with a time constant of 4.6 events. **(E–H)**, as **(A–D)**, respectively, except data are from a *Tsc1^+/-^* cell. Error bars indicate SEM.

**FIGURE 5:**
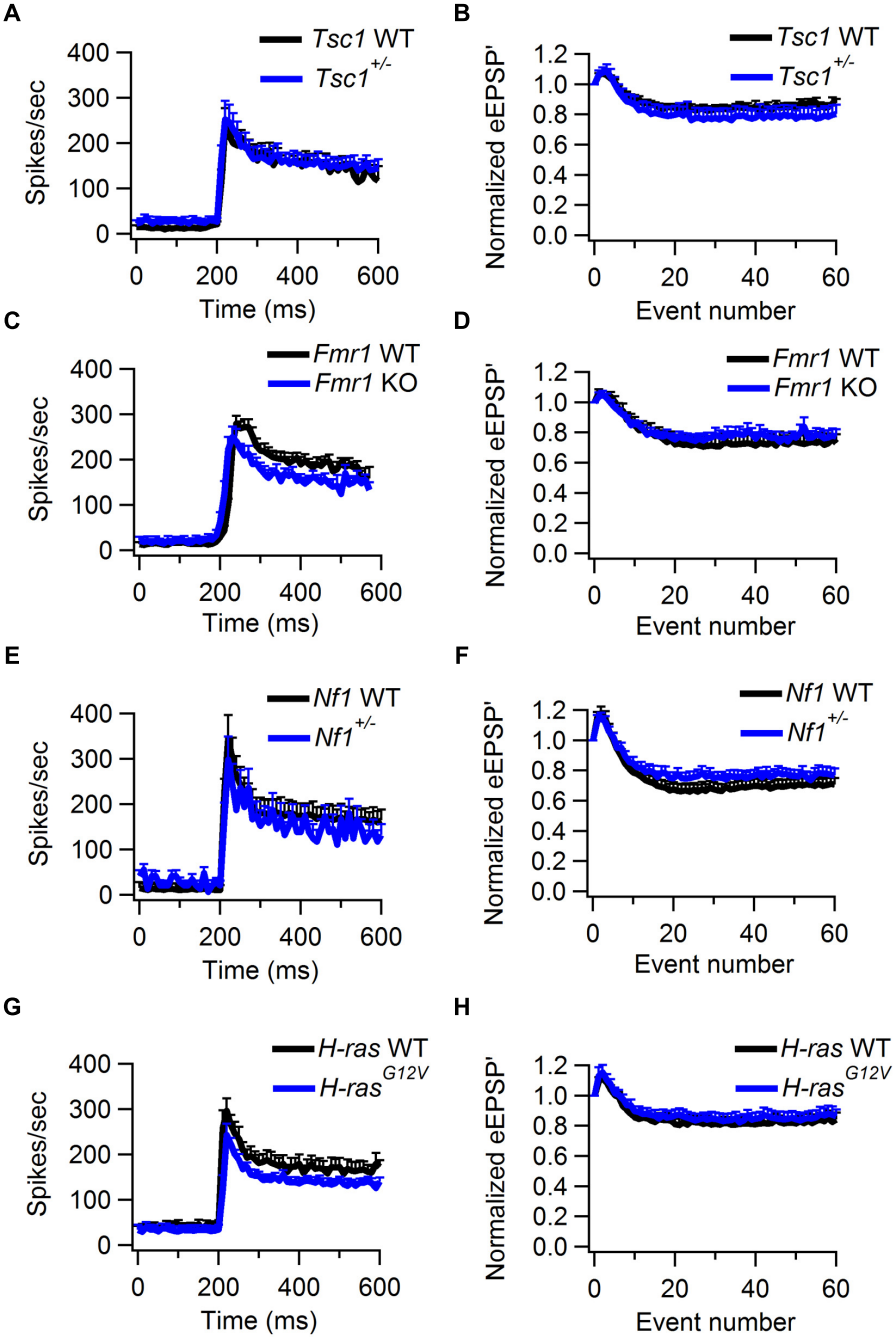
**Sound-evoked STD in WT and *Tsc1^+/-^*, *Fmr1* KO, *Nf1^+/-^*, and *H-ras^G12V^* mutant mice. (A)** Average peristimulus time histogram from WT (black) and *Tsc1^+/-^* mutants (blue). Error bars indicate SEM. **(B)** Normalized average of the amplitude of eEPSP’ against sound-evoked event number from recordings of WT (black) and *Tsc1^+/-^* animals (blue). Error bars indicate SEM. **(C,D)**, as **(A,B)**, except data is from WT and *Fmr1* KO animals. **(E,F)**, as **(A,B)**, except data is from WT and *Nf1^+/-^* animals. **(G,H)**, as **(A,B)**, except data is from WT and *H-ras^G12V^* animals.

No significant differences in synaptic transmission were observed in the other three lines. In *Tsc1*^+/-^ mice, the complex waveform had a similar shape as WT controls (**Figures 4A,E**; **Table 1**). Other aspects of the waveform did also not differ between *Tsc1*^+/-^ mice and their WT controls, although there was a tendency for the prespike-eAP delay to be shorter in the WT controls than in the *Tsc1*^+/-^ mice (0.55 ± 0.02 ms vs. 0.68 ± 0.04 ms). Presentation of a 400 ms, 80 dB noise burst elicited a clear firing increase (**Figures 4A,B,E,F**). Both spontaneous frequency, maximum evoked frequency and steady-state frequency were similar between *Tsc1*^+/-^ mice and their WT controls (**Figure 5A**; **Table 1**). *Fmr1* KO animals did not exhibit alterations in juxtacellular complex waveforms and firing behavior (**Table 1**; **Figure 5C**). Spontaneous, maximum evoked, and steady-state evoked frequencies were also not statistically different between *Nf1^+/-^* its WT controls (**Figure 5E**). *H-ras^G12V^* mutant mice and their respective WT littermates did not show any clear differences in the measured parameters (**Table 1**; **Figure 5G**).

**FIGURE 6:**
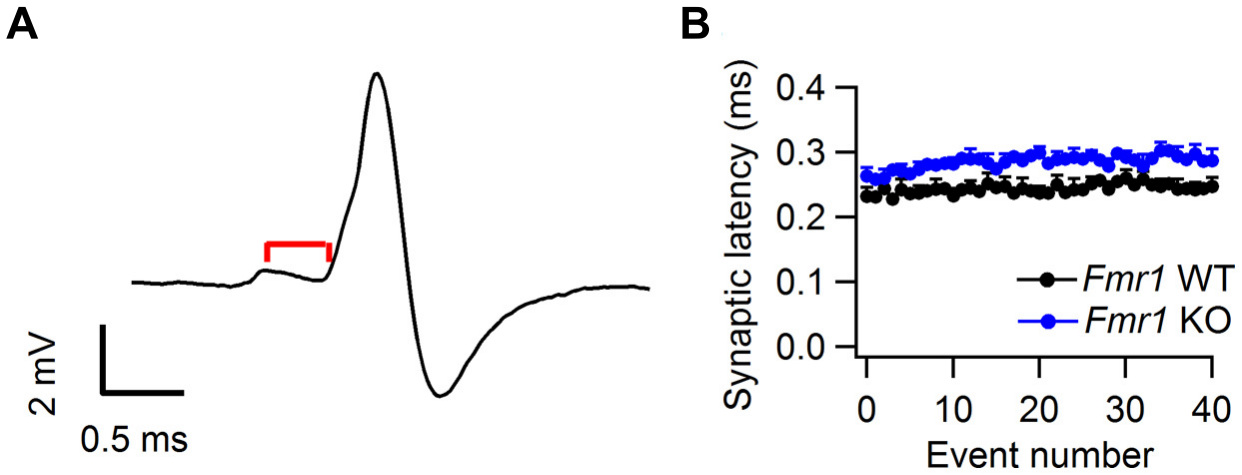
**Synaptic latency in *Fmr1* WT and KO mice. (A)** Representative complex waveform from a *Fmr1* WT neuron. Red line indicates the latency between prespike and EPSP onset **(B)** Relation between average synaptic latency and sound-evoked event number. Data is from both *Fmr1* WT (black) and *Fmr1* KO animals. Error bars indicate the SEM of the different averages.

In an earlier slice study reduced K_Na_ currents were found in the MNTB of *Fmr1* KO mice (Brown et al., 2010b). These channels are thought to improve temporal fidelity during high-frequency firing (Yang et al., 2007). We did not find a difference in prespike-AP latency between *Fmr1* KO mice and WT controls (**Table 1**), but to further evaluate possible differences during high-frequency signaling, we also measured the synaptic latency, which was defined as the latency between the peak of the prespike and the onset of the eEPSP (See Materials and Methods; **Figure 6A**). As shown in **Figure 6B**, the prespike-eEPSP delay changed only little after sound onset. *Fmr1* KO animals exhibited slightly longer synaptic latency than WT, but the difference did not reach significance (0.29 ± 0.01 ms in WT, *n* = 6 vs. 0.25 ± 0.01 ms in *Fmr1* KO, *n* = 5; *p* = 0.09).

### No Obvious Alterations in STP in the Mutant Animals *In Vivo*

We used the maximum of the first derivative of the extracellularly recorded EPSP (eEPSP’) from the characteristic complex waveform as a measure for the strength of synaptic transmission during auditory stimulation (**Figures 4A,C,D**). At sound onset, the amplitudes often showed a transient increase in both mutant and WT animals (**Figures 4C,G**). The ratio between the average amplitudes of the second sound-evoked eEPSP’ and the eEPSP’ before sound stimulation was used as an estimate for synaptic facilitation. The amount of facilitation was similar between *Tsc1^+/-^* and WT animals (**Figure 5B**; **Table 1**). At a later time point during the sound presentation, the average eEPSP’ in both WT and *Tsc1^+/-^* animals often decreased to a lower level, indicating that the high firing frequencies induced short-term STD (**Figures 4C,D,G,H, and 5B**). *Tsc1^+/-^* and WT animals showed similar levels of STD. To further investigate the STD of mutant and WT mice, we estimated the time course of STD during sound stimulation. To quantify how many events it took for the synapse to reach steady-state depression, the relation between the eEPSP’ and its event number following sound onset was plotted; the decay of the amplitudes could generally be well described by a single exponential function (**Figures 4D,H**), and we used the time constant of the single exponential fit as a measure for how rapidly the steady-state was reached. No obvious difference was observed in the time course or the extent of steady-state depression between *Tsc1^+/-^* and WT animals (**Figure 5B**; **Table 1**). Similar results were obtained for the other three lines (**Figures 5D,F,H**; **Table 1**).

**FIGURE 7:**
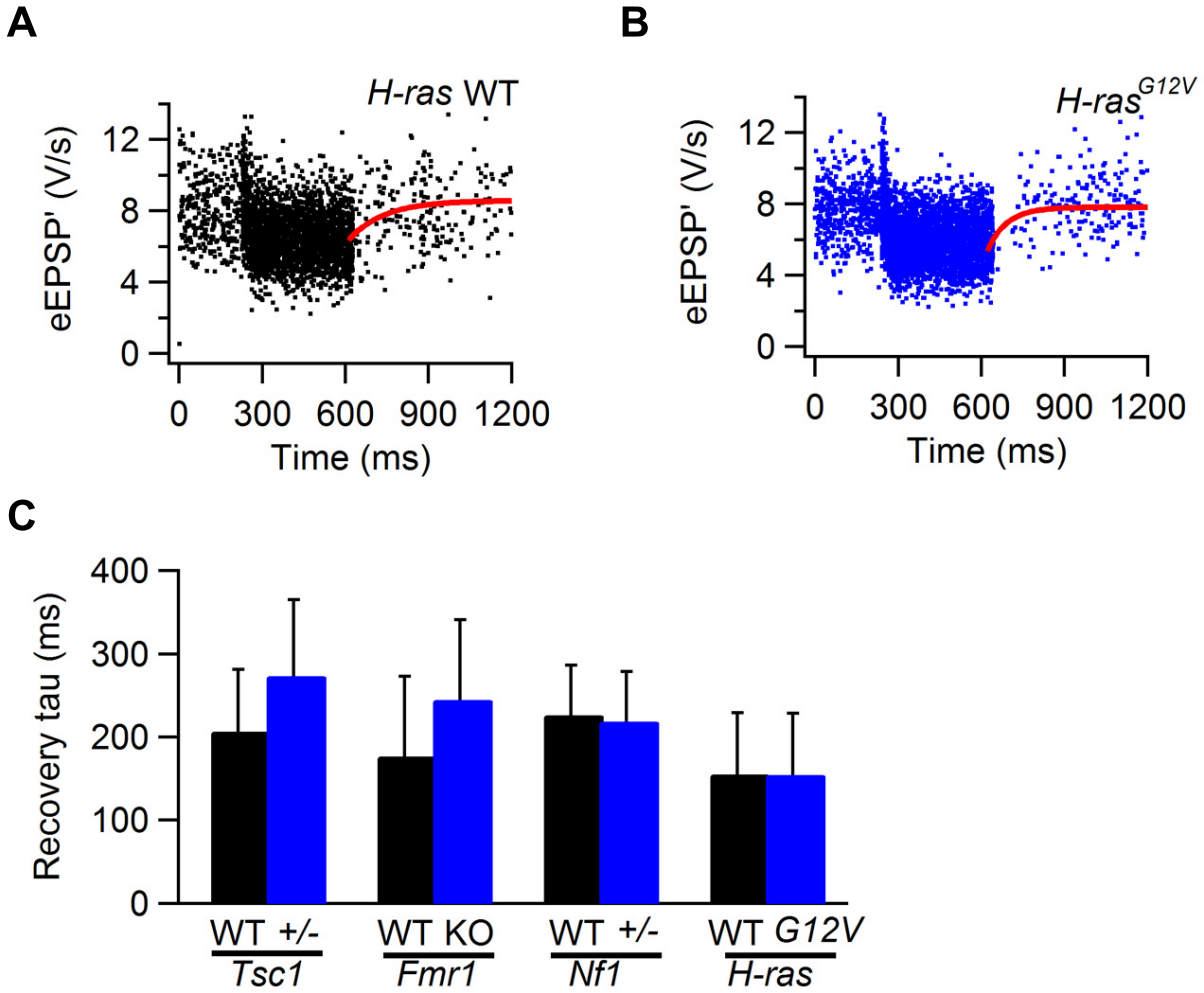
**Recovery from STD in WT and *Tsc1^+/-^*, *Fmr1* KO, *Nf1^+/-^*, and *H-ras^G12V^* mutant mice. (A)** Sound-evoked eEPSP’ in WT animal. Solid line (red) is fit of recovery from sound-evoked STD with single exponential function with time constant of 127 ms. **(B)** As **(A)**, except the recording is from a *H-ras^G12V^* animal. Fit time constant was 159 ms. **(C)** Comparison of the recovery time constants from WT (black) and *Tsc1^+/-^*, *Fmr1* KO, *Nf1^+/-^*, and *H-ras^G12V^* mutants. Error bars indicate SEM.

After auditory stimulation, the depressed eEPSP’ gradually recovered to their original level. Recovery from depression can generally be adequately described by a single exponential function, and the time constant generated by the fit can be used as a measure for the speed of recovery. Analysis was restricted to cells that showed >15% depression. As an example, recovery from STD in a cell from a *H-ras^G12V^* mouse and from its WT control are shown in **Figures 7A,B**. Recovery varied between cells, but overall did not differ appreciably between mutant and WT cells in all four lines (**Figure 7C**; **Table 1**).

## Discussion

The aim of this study was to investigate how dysregulation of mTOR and/or Ras signaling in neurogenetic disorders affects synaptic transmission. To address this question, we compared the morphology, firing behavior, AP waveforms, and STP in the calyx of Held synapse of *Tsc1^+/-^*, *Fmr1* KO, *Nf1^+/-^*, and *H-ras^G12V^* mice with their respective WT littermates. Increased volume and surface area were observed in *Tsc1^+/-^* and in *Fmr1* KO mice, and calyces of *Fmr1* KO mice also showed more complex morphology. In addition we found a slightly shorter delay between EPSPs and APs in the *Nf1^+/-^* mice than in their WT controls.

### Comparison with Earlier Results

The calyx of Held morphology of the WT controls in the present study matched our previous study of the *Cacna1a* mutant (Di Guilmi et al., 2014). Type I calyces were found in none of the young-adult mice in the present and previous studies, which differs from the reported calyx morphology in two previous studies (Grande and Wang, 2011; Grande et al., 2014). Possibly, a developmental difference is responsible for this, since these previous studies focused on P16–P19 mice, whereas in the present study and our two earlier studies, young-adult mice were used.

Spontaneous firing rates in this study were lower than in our earlier study in the C57BL/6 background (Lorteije et al., 2009), but similar as in other studies in both mice and other species (Kopp-Scheinpflug et al., 2008; Wang et al., 2013). Synaptic transmission along the calyx of Held synapse in the WT animals generally matched our previous *in vivo* experiments as well, including the observation that STD during a tone was lower in the studies in which spontaneous frequency was higher, presumably because of larger tonic depression (Lorteije et al., 2009; Wang et al., 2013).

At high spike rates, the synaptic delay of the calyx can increase considerably (Guinan and Li, 1990; Fedchyshyn and Wang, 2007; Kim et al., 2007; Mc Laughlin et al., 2008; Tolnai et al., 2009). However, we found that the prespike-eEPSP delay changed only little after sound onset, in agreement with the role of the calyx of Held synapse as an auditory relay (Borst and Soria van Hoeve, 2012). In previous *in vivo* studies the increase appeared to be mainly in the delay between EPSP and AP (Guinan and Li, 1990; Mc Laughlin et al., 2008; Tolnai et al., 2009), in line with a role of spike depression at high frequencies (Lorteije et al., 2009). The experiments in which an increase in the delay between prespike and EPSP onset was observed in slice recordings were performed in juvenile animals (Fedchyshyn and Wang, 2007; Kim et al., 2007), therefore maturational changes may have contributed to this difference with our results.

### Tuberous Sclerosis Complex

We observed that calyces were larger in *Tsc1^+/-^* mice, but that synaptic transmission was not different from WT littermates. Similar findings have been obtained in the *Drosophila* neuromuscular junction (Knox et al., 2007; Natarajan et al., 2013). In *Drosophila* overexpression of Rheb in motoneurons and the resulting Tor activation produces profound synaptic overgrowth (Knox et al., 2007). Absence of either Tsc1 or Tsc2 also resulted in increased synapse growth. However, despite an increase in the number of synaptic boutons and active zones, quantal content was not changed in the absence of Tsc2 in the *Drosophila* neuromuscular junction (Natarajan et al., 2013). In rodents, even though loss-of-function mutations in *Tsc1* or *Tsc2* are known to alter excitatory synapse structure, these changes may to a large extent be secondary to altered network activity, as no morphological changes were observed if *Tsc1* was deleted in isolated neurons *in vivo* (Bateup et al., 2011). The largest effects on excitatory transmission also appear to be secondary to network hyperactivity. Variable effects on APs have been reported in neurons that lack TSC1 (Bateup et al., 2013; Normand et al., 2013; Weston et al., 2014), but no net change in glutamatergic synapse-driven excitability in isolated *Tsc1* KO neurons, autaptic hippocampal neurons, or Purkinje cells was observed (Tsai et al., 2012; Bateup et al., 2013; Weston et al., 2014), although a variety of changes can be observed due to hyperactivity (Tavazoie et al., 2005). In *Tsc2*^+/^*^-^* mice, field recordings showed normal basal synaptic transmission and paired-pulse facilitation at the Schaffer collateral–CA1 synapse (Ehninger et al., 2008). In mice in which *Tsc1* was acutely deleted in the adult, hippocampal neurons showed normal basal synaptic transmission, but increased excitability and maximal firing rate (Abs et al., 2013).

An important synaptic phenotype in TSC rodent models are changes in long-term plasticity, which have been observed in the *Tsc2^+/-^* Eker rat (von der Brelie et al., 2006), *Tsc2*^+/^*^-^* mice (Ehninger et al., 2008), and in the absence of *Tsc1* in mice (Bateup et al., 2011; Abs et al., 2013). A comparison with the calyx of Held synapse is not possible, since there are currently no known protocols to induce forms of long-term plasticity in the adult calyx of Held synapse.

### Fragile X Syndrome

Similar to the *Tsc1^+/-^* mice, the *Fmr1* KO mice also showed larger calyces. Moreover, a larger fraction of the calyces from *Fmr1* KO animals had a large number of boutons (>15) compared to WT. FMRP has been shown to be present in presynaptic terminals (Christie et al., 2009), and enlarged synaptic terminals were also observed in a *Drosophila* FXS model (Zhang et al., 2001). Besides its presynaptic function, FMRP is known as an important translational regulator in postsynaptic compartments. Loss of FMRP is linked to dysregulation of local protein synthesis, which could result in disrupted metabotropic glutamate receptor signaling, as well as long-term synaptic plasticity deficits (Huber et al., 2002).

The lack of a functional synaptic phenotype in the *Fmr1* KO mice was unexpected, considering that many presynaptic effects on STP are mediated by a direct effect of FMRP on BK channels (Deng et al., 2013; Myrick et al., 2015), and these channels are also present in the calyx of Held (Nakamura and Takahashi, 2007). Why we observed no effect on synaptic strength or STP is therefore presently not clear. The lack of an effect on recovery from STD is in agreement with results from hippocampal neurons (Deng et al., 2011). Part of the presynaptic effects of FMRP occur via a direct interaction with N-type (Ca_V_2.2) Ca channels (Ferron et al., 2014). These channels are not present in the adult calyx of Held (Iwasaki et al., 2000), which could be another reason for the lack of a presynaptic phenotype in our study compared to earlier studies in the hippocampus.

Fragile X syndrome mouse models show a variety of abnormalities in auditory processing (Rotschafer and Razak, 2014). Two studies in the MNTB have shown that FMRP affects potassium channels. FMRP binds to the *Slack* sodium-activated potassium channel (K_Na_) to activate the channel, and a slice study of principal neurons in the MNTB showed that a reduced fraction of potassium conductance is carried by K_Na_ currents in the *Fmr1* KO mice (Brown et al., 2010b). These channels are thought to improve temporal fidelity during high-frequency firing (Yang et al., 2007). We observed no obvious changes in fidelity, maximal firing rate, prespike-eAP latency, or eAP halfwidth in the *Fmr1* KO mice. There was a tendency for synaptic latencies (defined as the latency between prespike and eEPSP onset) to be longer in the *Fmr1* KO mice, but this difference did not reach significance. Possible explanations for the lack of a change in temporal fidelity in our *in vivo* experiments include age difference (slice studies were done on P9–P14 animals), strain difference, and an insufficiently high firing frequency *in vivo*. Another slice study from the same group showed less of an increase in the Kv3.1 currents in the medial (high-frequency) region in the *Fmr1* KO mice (Strumbos et al., 2010). In our experiments we did not specifically target the medial part, so we may have easily missed a decrease in the tonotopic gradient for these channels. Phenotypic responses in FXS mouse models have been shown to depend on genetic background (Kooy, 2003; Spencer et al., 2011). Even though auditory phenotypes have typically been demonstrated in C57BL/6J mice, the background studied here, we cannot exclude that a phenotype would have been found in another background, that genetic drift contributed to the lack of a phenotype, or that the difference in the background used in the earlier MNTB studies (FVB vs. C57BL/6J) contributed to the lack of a difference on waveforms.

### Neurofibromatosis Type 1

We observed evidence for a decrease in the delay between the EPSP and the AP in the *Nf1*^+/^*^-^* mice. The effect was not very large, but did reach significance. Both pre- and postsynaptic factors could contribute to this change. An increased release probability seems less likely at present, since this would probably be accompanied by increased STD during the tone presentation, which was not observed. We cannot exclude that changes in inhibitory inputs to the principal neurons contribute to the apparent change in EPSP-AP coupling in the *Nf1*^+/^*^-^* mice, but there is little evidence for a strong contribution of synaptic inhibition to the complex extracellular waveform at the level of the mouse MNTB (Lorteije and Borst, 2011). In both dorsal root ganglion neurons and hippocampal or neocortical interneurons from NF1 mice clear increases in excitability have been observed (Wang et al., 2005; Omrani et al., 2015). In the dorsal root ganglion neurons the increased excitability is caused by increased sodium conductance, which depends on the increased Ras activity (Wang et al., 2010; Duan et al., 2011). In the interneurons these changes in excitability were caused by a decrease in the hyperpolarization-activated non-selective cation current *I*_h_ (Omrani et al., 2015), which is also prominently expressed in the principal neurons of the MNTB (Banks et al., 1993; Leao et al., 2006). Interestingly, these changes in the interneurons were not observed in the *H-Ras^G12V^* knock-in mice, even though these mice have even stronger increase in Ras-ERK signaling than NF1 mice (Omrani et al., 2015). As we did not observe changes in EPSP-AP coupling in the *H-Ras^G12V^* mice, one can speculate that the changes in the *Nf1*^+/^*^-^* mice are also caused by a direct interaction of NF1 with HCN channels. However, *in vivo* whole-cell recordings and slice recordings will be needed to confirm the observed changes of the present study, and to test whether a change in *I*_h_ leading to a more hyperpolarized membrane potential and increased membrane resistance is underlying the apparent increased excitability (Omrani et al., 2015).

### Costello Syndrome

Little is currently known about possible changes in synaptic transmission in CS (Stornetta and Zhu, 2011). Expression of the *H-Ras^G12V^* transgene in excitatory neurons of the hippocampus results in a higher density of docked neurotransmitter vesicles in glutamatergic terminals, an increased frequency of miniature EPSCs, and increased paired-pulse facilitation (Kushner et al., 2005). However, we did not find evidence for similar changes at the calyx of Held in the CS mouse model, in which the G12V mutation is inserted in the endogenous *H-Ras* allele. Ras signaling in pyramidal neurons is required for many forms of long-term synaptic plasticity. *H-ras* KO mice show increased NMDAR-dependent synaptic transmission (Manabe et al., 2000). Changes in NMDAR signaling would not be easy to detect in the adult calyx of Held synapse, since this receptor is downregulated in the MNTB after hearing onset (Taschenberger and von Gersdorff, 2000; Futai et al., 2001). Ras is also important for controlling synaptic AMPAR delivery during long-term plasticity (Zhu et al., 2002), but, as discussed above, testing for a change in LTP in the MNTB of the CS mouse model is currently not possible.

## Conclusion

Here, we have tested four commonly used mouse models for hereditary forms of intellectual disability for changes in presynaptic morphology, baseline synaptic transmission, and short-term plasticity at the calyx of Held synapse. Changes in morphology in the mouse models for TSC and FXS were not reflected in obvious changes in baseline synaptic transmission or short-term plasticity. This is not without precedent, since pronounced morphological changes without changes in synaptic transmission were observed following deletion of an exocyst subunit at the calyx synapse (Schwenger and Kuner, 2010). Possibly, homeostatic changes may be responsible for the lack of changes in synaptic transmission in the TSC and FXS mouse models, but more detailed analysis of for example the number of active zones in these mice would first be needed before this could be further investigated. We found some evidence that EPSP-AP coupling was stronger in the *NF1^+/-^* mice, but this was not observed in the CS mouse, which is like NF1 a RASopathy (Rauen, 2013). Even though many forms of intellectual disability are thought to be due to changes in synaptic transmission (Swiech et al., 2008; Stornetta and Zhu, 2011; Levenga and Willemsen, 2012), our results thus show that changes in mTOR or Ras signaling do not result in ubiquitous *in vivo* changes in synaptic transmission.

## Author Contributions

TW and LK did experiments. TW, LK, GB analyzed data. GB wrote analysis software. TW, GB wrote the Ms. RW, YE contributed materials. All authors contributed to the planning of the experiments, commented on earlier versions of the MS, and approved the final version of the Ms.

## Conflict of Interest Statement

The authors declare that the research was conducted in the absence of any commercial or financial relationships that could be construed as a potential conflict of interest.

## References

[B1] Abs E. Goorden S. M. I. Schreiber J. Overwater I. E. Hoogeveen-Westerveld M. Bruinsma C. F. (2013). TORC1-dependent epilepsy caused by acute biallelic Tsc1 deletion in adult mice. *Ann. Neurol.* 74 569–579. 10.1002/ana.23943 23720219

[B2] Acebes A. Ferrús A. (2001). Increasing the number of synapses modifies olfactory perception in *Drosophila*. *J. Neurosci.* 21 6264–6273. 11487649 10.1523/JNEUROSCI.21-16-06264.2001 PMC6763191

[B3] Aoki Y. Niihori T. Kawame H. Kurosawa K. Ohashi H. Tanaka Y. (2005). Germline mutations in HRAS proto-oncogene cause Costello syndrome. *Nat. Genet.* 37 1038–1040. 10.1038/ng1641 16170316

[B4] Ascano M. Jr. Mukherjee N. Bandaru P. Miller J. B. Nusbaum J. D. Corcoran D. L. (2012). FMRP targets distinct mRNA sequence elements to regulate protein expression. *Nature* 492 382–386. 10.1038/nature11737 23235829 PMC3528815

[B5] Axelrad M. E. Schwartz D. D. Katzenstein J. M. Hopkins E. Gripp K. W. (2011). Neurocognitive, adaptive, and behavioral functioning of individuals with Costello syndrome: a review. *Am. J. Med. Genet. C Semin. Med. Genet.* 157C, 115–122. 10.1002/ajmg.c.30299 21495179

[B6] Bakker C. E. De Diego Otero Y. Bontekoe C. Raghoe P. Luteijn T. Hoogeveen A. T. (2000). Immunocytochemical and biochemical characterization of FMRP, FXR1P, and FXR2P in the mouse. *Exp. Cell Res.* 258 162–170. 10.1006/excr.2000.4932 10912798

[B7] Banks M. I. Pearce R. A. Smith P. H. (1993). Hyperpolarization-activated cation current (*I*_h_) in neurons of the medial nucleus of the trapezoid body: voltage-clamp analysis and enhancement by norepinephrine and cAMP suggest a modulatory mechanism in the auditory brain stem. *J. Neurophysiol.* 70 1420–1432. 7506755 10.1152/jn.1993.70.4.1420

[B8] Basu T. N. Gutmann D. H. Fletcher J. A. Glover T. W. Collins F. S. Downward J. (1992). Aberrant regulation of ras proteins in malignant tumour cells from type 1 neurofibromatosis patients. *Nature* 356 713–715. 10.1038/356713a0 1570015

[B9] Bateup H. S. Johnson C. A. Denefrio C. L. Saulnier J. L. Kornacker K. Sabatini B. L. (2013). Excitatory/inhibitory synaptic imbalance leads to hippocampal hyperexcitability in mouse models of tuberous sclerosis. *Neuron* 78 510–522. 10.1016/j.neuron.2013.03.017 23664616 PMC3690324

[B10] Bateup H. S. Takasaki K. T. Saulnier J. L. Denefrio C. L. Sabatini B. L. (2011). Loss of Tsc1 in vivo impairs hippocampal mGluR-LTD and increases excitatory synaptic function. *J. Neurosci.* 31 8862–8869. 10.1523/JNEUROSCI.1617-11.2011 21677170 PMC3133739

[B11] Batista P. B. Lemos S. M. A. Rodrigues L. O. C. De Rezende N. A. (2014). Auditory temporal processing deficits and language disorders in patients with neurofibromatosis type 1. *J. Commun. Disord.* 48 18–26. 10.1016/j.jcomdis.2013.12.002 24447521

[B12] Bhakar A. L. Dölen G. Bear M. F. (2012). The pathophysiology of fragile X (and what it teaches us about synapses). *Annu. Rev. Neurosci.* 35 417–443. 10.1146/annurev-neuro-060909-153138 22483044 PMC4327822

[B13] Borst J. G. G. Helmchen F. Sakmann B. (1995). Pre- and postsynaptic whole-cell recordings in the medial nucleus of the trapezoid body of the rat. *J. Physiol.* 489 825–840. 10.1113/jphysiol.1995.sp021095 8788946 PMC1156851

[B14] Borst J. G. G. Soria van Hoeve J. (2012). The calyx of Held synapse: from model synapse to auditory relay. *Annu. Rev. Physiol.* 74 199–224. 10.1146/annurev-physiol-020911-153236 22035348

[B15] Boyle L. Kaufmann W. E. (2010). The behavioral phenotype of FMR1 mutations. *Am. J. Med. Genet. C Semin. Med. Genet.* 154C, 469–476. 10.1002/ajmg.c.30277 20981777

[B16] Brager D. H. Johnston D. (2014). Channelopathies and dendritic dysfunction in fragile X syndrome. *Brain Res. Bull.* 103 11–17. 10.1016/j.brainresbull.2014.01.002 24462643 PMC4049233

[B17] Brown J. A. Gianino S. M. Gutmann D. H. (2010a). Defective cAMP generation underlies the sensitivity of CNS neurons to neurofibromatosis-1 heterozygosity. *J. Neurosci.* 30 5579–5589. 10.1523/JNEUROSCI.3994-09.2010 20410111 PMC2864934

[B18] Brown M. R. Kronengold J. Gazula V.-R. Chen Y. Strumbos J. G. Sigworth F. J. (2010b). Fragile X mental retardation protein controls gating of the sodium-activated potassium channel Slack. *Nat. Neurosci.* 13 819–821. 10.1038/nn.2563 20512134 PMC2893252

[B19] Canal I. Acebes A. Ferrús A. (1998). Single neuron mosaics of the *Drosophila gigas* mutant project beyond normal targets and modify behavior. *J. Neurosci.* 18 999–1008. 9437021 10.1523/JNEUROSCI.18-03-00999.1998 PMC6792781

[B20] Carvill S. (2001). Sensory impairments, intellectual disability and psychiatry. *J. Intellect. Disabil. Res.* 45 467–483. 10.1046/j.1365-2788.2001.00366.x 11737534

[B21] Christie S. B. Akins M. R. Schwob J. E. Fallon J. R. (2009). The FXG: a presynaptic fragile X granule expressed in a subset of developing brain circuits. *J. Neurosci.* 29 1514–1524. 10.1523/JNEUROSCI.3937-08.2009 19193898 PMC2746438

[B22] Corbin F. Bouillon M. Fortin A. Morin S. Rousseau F. Khandjian E. W. (1997). The fragile X mental retardation protein is associated with poly(A)^+^ mRNA in actively translating polyribosomes. *Hum. Mol. Genet.* 6 1465–1472. 10.1093/hmg/6.9.1465 9285783

[B23] Costa R. M. Federov N. B. Kogan J. H. Murphy G. G. Stern J. Ohno M. (2002). Mechanism for the learning deficits in a mouse model of neurofibromatosis type 1. *Nature* 415 526–530. 10.1038/nature711 11793011

[B24] Crino P. B. (2011). mTOR: a pathogenic signaling pathway in developmental brain malformations. *Trends Mol. Med.* 17 734–742. 10.1016/j.molmed.2011.07.008 21890410

[B25] Crins T. T. H. Rusu S. I. Rodríguez-Contreras A. Borst J. G. G. (2011). Developmental changes in short-term plasticity at the rat calyx of Held synapse. *J. Neurosci.* 31 11706–11717. 10.1523/JNEUROSCI.1995-11.2011 21832200 PMC4314708

[B26] Cui Y. Costa R. M. Murphy G. G. Elgersma Y. Zhu Y. Gutmann D. H. (2008). Neurofibromin regulation of ERK signaling modulates GABA release and learning. *Cell* 135 549–560. 10.1016/j.cell.2008.09.060 18984165 PMC2673196

[B27] Daston M. M. Ratner N. (1992). Neurofibromin, a predominantly neuronal GTPase activating protein in the adult, is ubiquitously expressed during development. *Dev. Dyn.* 195 216–226. 10.1002/aja.1001950307 1301085

[B28] DeClue J. E. Cohen B. D. Lowy D. R. (1991). Identification and characterization of the neurofibromatosis type 1 protein product. *Proc. Natl. Acad. Sci. U.S.A.* 88 9914–9918. 10.1073/pnas.88.22.9914 1946460 PMC52837

[B29] DeClue J. E. Papageorge A. G. Fletcher J. A. Diehl S. R. Ratner N. Vass W. C. (1992). Abnormal regulation of mammalian p21ras contributes to malignant tumor growth in von Recklinghausen (type 1) neurofibromatosis. *Cell* 69 265–273. 10.1016/0092-8674(92)90407-4 1568246

[B30] Deng P. Y. Rotman Z. Blundon J. A. Cho Y. Cui J. Cavalli V. (2013). FMRP regulates neurotransmitter release and synaptic information transmission by modulating action potential duration via BK channels. *Neuron* 77 696–711. 10.1016/j.neuron.2012.12.018 23439122 PMC3584349

[B31] Deng P.-Y. Sojka D. Klyachko V. A. (2011). Abnormal presynaptic short-term plasticity and information processing in a mouse model of fragile X syndrome. *J. Neurosci.* 31 10971–10982. 10.1523/JNEUROSCI.2021-11.2011 21795546 PMC6623101

[B32] Di Guilmi M. N. Wang T. Inchauspe C. G. Forsythe I. D. Ferrari M. D. Van Den Maagdenberg A. M. J. M. (2014). Synaptic gain-of-function effects of mutant Cav2.1 channels in a mouse model of familial hemiplegic migraine are due to increased basal [Ca^2+^]_i_. *J. Neurosci.* 34 7047–7058. 10.1523/jneurosci.2526-13.2014 24849341 PMC4028489

[B33] Duan J. H. Wang Y. Duarte D. Vasko M. R. Nicol G. D. Hingtgen C. M. (2011). Ras signaling pathways mediate NGF-induced enhancement of excitability of small-diameter capsaicin-sensitive sensory neurons from wildtype but not Nf1^+/-^ mice. *Neurosci. Lett.* 496 70–74. 10.1016/j.neulet.2011.03.083 21501659 PMC3101079

[B34] Ebrahimi-Fakhari D. Sahin M. (2015). Autism and the synapse: emerging mechanisms and mechanism-based therapies. *Curr. Opin. Neurol.* 28 91–102. 10.1097/wco.0000000000000186 25695134

[B35] Ehninger D. Han S. Shilyansky C. Zhou Y. Li W. Kwiatkowski D. J. (2008). Reversal of learning deficits in a Tsc2^+/-^ mouse model of tuberous sclerosis. *Nat. Med.* 14 843–848. 10.1038/nm1788 18568033 PMC2664098

[B36] Fedchyshyn M. J. Wang L.-Y. (2007). Activity-dependent changes in temporal components of neurotransmission at the juvenile mouse calyx of Held synapse. *J. Physiol.* 581 581–602. 10.1113/jphysiol.2007.129833 17347264 PMC2075169

[B37] Feng Y. Absher D. Eberhart D. E. Brown V. Malter H. E. Warren S. T. (1997). FMRP associates with polyribosomes as an mRNP, and the I304N mutation of severe fragile X syndrome abolishes this association. *Mol. Cell* 1 109–118. 10.1016/S1097-2765(00)80012-X 9659908

[B38] Ferron L. Nieto-Rostro M. Cassidy J. S. Dolphin A. C. (2014). Fragile X mental retardation protein controls synaptic vesicle exocytosis by modulating N-type calcium channel density. *Nat. Commun.* 5 3628 10.1038/ncomms4628 PMC3982139 24709664

[B39] Forsythe I. D. (1994). Direct patch recording from identified presynaptic terminals mediating glutamatergic EPSCs in the rat CNS, in vitro. *J. Physiol.* 479 381–387. 10.1113/jphysiol.1994.sp020303 7837096 PMC1155757

[B40] Frankland P. W. Wang Y. Rosner B. Shimizu T. Balleine B. W. Dykens E. M. (2004). Sensorimotor gating abnormalities in young males with fragile X syndrome and Fmr1-knockout mice. *Mol. Psychiatry* 9 417–425. 10.1038/sj.mp.4001432 14981523

[B41] Freygang W. H. Jr. Frank K. (1959). Extracellular potentials from single spinal motoneurons. *J. Gen. Physiol.* 42 749–760. 10.1085/jgp.42.4.749 13631201 PMC2195006

[B42] Furth M. E. Aldrich T. H. Cordon-Cardo C. (1987). Expression of ras proto-oncogene proteins in normal human tissues. *Oncogene* 1 47–58. 3125507

[B43] Futai K. Okada M. Matsuyama K. Takahashi T. (2001). High-fidelity transmission acquired via a developmental decrease in NMDA receptor expression at an auditory synapse. *J. Neurosci.* 21 3342–3349. 11331363 10.1523/JNEUROSCI.21-10-03342.2001 PMC6762464

[B44] Grande G. Negandhi J. Harrison R. V. Wang L. Y. (2014). Remodelling at the calyx of Held-MNTB synapse in mice developing with unilateral conductive hearing loss. *J. Physiol.* 592 1581–1600. 10.1113/jphysiol.2013.268839 24469075 PMC3979613

[B45] Grande G. Wang L.-Y. (2011). Morphological and functional continuum underlying heterogeneity in the spiking fidelity at the calyx of Held synapse in vitro. *J. Neurosci.* 31 13386–13399. 10.1523/JNEUROSCI.0400-11.2011 21940432 PMC6623283

[B46] Gross C. Nakamoto M. Yao X. Chan C. B. Yim S. Y. Ye K. (2010). Excess phosphoinositide 3-kinase subunit synthesis and activity as a novel therapeutic target in fragile X syndrome. *J. Neurosci.* 30 10624–10638. 10.1523/jneurosci.0402-10.2010 20702695 PMC2924772

[B47] Guinan J. J. Jr. Li R. Y.-S. (1990). Signal processing in brainstem auditory neurons which receive giant endings (calyces of Held) in the medial nucleus of the trapezoid body of the cat. *Hear. Res.* 49 321–334. 10.1016/0378-5955(90)90111-2 2292504

[B48] Gutmann D. H. Wood D. L. Collins F. S. (1991). Identification of the neurofibromatosis type 1 gene product. *Proc. Natl. Acad. Sci. U.S.A.* 88 9658–9662. 10.1073/pnas.88.21.9658 1946382 PMC52777

[B49] Gutmann D. H. Zhang Y. Hasbani M. J. Goldberg M. P. Plank T. L. Petri Henske E. (2000). Expression of the tuberous sclerosis complex gene products, hamartin and tuberin, in central nervous system tissues. *Acta Neuropathol.* 99 223–230. 10.1007/PL00007431 10663963

[B50] Huber K. M. Gallagher S. M. Warren S. T. Bear M. F. (2002). Altered synaptic plasticity in a mouse model of fragile X mental retardation. *Proc. Natl. Acad. Sci. U.S.A.* 99 7746–7750. 10.1073/pnas.122205699 12032354 PMC124340

[B51] Iwasaki S. Momiyama A. Uchitel O. D. Takahashi T. (2000). Developmental changes in calcium channel types mediating central synaptic transmission. *J. Neurosci.* 20 59–65. 10627581 10.1523/JNEUROSCI.20-01-00059.2000 PMC6774098

[B52] Jacks T. Shih T. S. Schmitt E. M. Bronson R. T. Bernards A. Weinberg R. A. (1994). Tumour predisposition in mice heterozygous for a targeted mutation in Nf1. *Nat. Genet.* 7 353–361. 10.1038/ng0794-353 7920653

[B53] Kassai H. Sugaya Y. Noda S. Nakao K. Maeda T. Kano M. (2014). Selective activation of mTORC1 signaling recapitulates microcephaly, tuberous sclerosis, and neurodegenerative diseases. *Cell Rep.* 7 1626–1639. 10.1016/j.celrep.2014.04.048 24857653

[B54] Kawame H. Matsui M. Kurosawa K. Matsuo M. Masuno M. Ohashi H. (2003). Further delineation of the behavioral and neurologic features in Costello syndrome. *Am. J. Med. Genet. A* 118A, 8–14. 10.1002/ajmg.a.10236 12605434

[B55] Kim J. H. Sizov I. Dobretsov M. Von Gersdorff H. (2007). Presynaptic Ca^2+^ buffers control the strength of a fast post-tetanic hyperpolarization mediated by the α3 Na^+^/K^+^-ATPase. *Nat. Neurosci.* 10 196–205. 10.1038/nn1839 17220883

[B56] Knox S. Ge H. Dimitroff B. D. Ren Y. Howe K. A. Arsham A. M. (2007). Mechanisms of TSC-mediated control of synapse assembly and axon guidance. *PLoS ONE* 2:e375 10.1371/journal.pone.0000375 PMC1847706 17440611

[B57] Kooy R. F. (2003). Of mice and the fragile X syndrome. *Trends Genet.* 19 148–154. 10.1016/S0168-9525(03)00017-9 12615009

[B58] Kopp-Scheinpflug C. Tolnai S. Malmierca M. S. Rübsamen R. (2008). The medial nucleus of the trapezoid body: comparative physiology. *Neuroscience* 154 160–170. 10.1016/j.neuroscience.2008.01.088 18436383

[B59] Krab L. C. Goorden S. M. Elgersma Y. (2008). Oncogenes on my mind: ERK and MTOR signaling in cognitive diseases. *Trends Genet.* 24 498–510. 10.1016/j.tig.2008.07.005 18774199

[B60] Kushner S. A. Elgersma Y. Murphy G. G. Jaarsma D. Van Woerden G. M. Hojjati M. R. (2005). Modulation of presynaptic plasticity and learning by the H-ras/extracellular signal-regulated kinase/synapsin I signaling pathway. *J. Neurosci.* 25 9721–9734. 10.1523/JNEUROSCI.2836-05.2005 16237176 PMC2802213

[B61] Lasarge C. L. Danzer S. C. (2014). Mechanisms regulating neuronal excitability and seizure development following mTOR pathway hyperactivation. *Front. Mol. Neurosci.* 7:18 10.3389/fnmol.2014.00018 PMC3953715 24672426

[B62] Leao K. E. Leao R. N. Sun H. Fyffe R. E. W. Walmsley B. (2006). Hyperpolarization-activated currents are differentially expressed in brainstem auditory nuclei. *J. Physiol.* 576(Pt 3), 849–864. 10.1113/jphysiol.2006.114702 16916913 PMC1890420

[B63] Levenga J. Willemsen R. (2012). Perturbation of dendritic protrusions in intellectual disability. *Prog. Brain Res.* 197 153–168. 10.1016/B978-0-444-54299-1.00008-X 22541292

[B64] Lorteije J. A. M. Borst J. G. G. (2011). Contribution of the mouse calyx of Held synapse to tone adaptation. *Eur. J. Neurosci.* 33 251–258. 10.1111/j.1460-9568.2010.07507.x 21198978

[B65] Lorteije J. A. M. Rusu S. I. Kushmerick C. Borst J. G. G. (2009). Reliability and precision of the mouse calyx of Held synapse. *J. Neurosci.* 29 13770–13784. 10.1523/JNEUROSCI.3285-09.2009 19889989 PMC6666705

[B66] Ma A. Wang L. Gao Y. Chang Z. Peng H. Zeng N. (2014). Tsc1 deficiency-mediated mTOR hyperactivation in vascular endothelial cells causes angiogenesis defects and embryonic lethality. *Hum. Mol. Genet.* 23 693–705. 10.1093/hmg/ddt456 24129405

[B67] Manabe T. Aiba A. Yamada A. Ichise T. Sakagami H. Kondo H. (2000). Regulation of long-term potentiation by H-Ras through NMDA receptor phosphorylation. *J. Neurosci.* 20 2504–2511. 10729330 10.1523/JNEUROSCI.20-07-02504.2000 PMC6772257

[B68] Mc Laughlin M. Van Der Heijden M. Joris P. X. (2008). How secure is in vivo synaptic transmission at the calyx of Held? *J. Neurosci.* 28 10206–10219. 10.1523/JNEUROSCI.2735-08.2008 18842881 PMC6671020

[B69] Meikle L. Mcmullen J. R. Sherwood M. C. Lader A. S. Walker V. Chan J. A. (2005). A mouse model of cardiac rhabdomyoma generated by loss of Tsc1 in ventricular myocytes. *Hum. Mol. Genet.* 14 429–435. 10.1093/hmg/ddi039 15601645

[B70] Mientjes E. J. Nieuwenhuizen I. Kirkpatrick L. Zu T. Hoogeveen-Westerveld M. Severijnen L. (2006). The generation of a conditional Fmr1 knock out mouse model to study Fmrp function *in vivo*. *Neurobiol. Dis.* 21 549–555. 10.1016/j.nbd.2005.08.019 16257225

[B71] Miller L. J. Mcintosh D. N. Mcgrath J. Shyu V. Lampe M. Taylor A. K. (1999). Electrodermal responses to sensory stimuli in individuals with fragile X syndrome: a preliminary report. *Am. J. Med. Genet.* 83 268–279. 10.1002/(SICI)1096-8628(19990402)83:4<268::AID-AJMG7>3.0.CO;2-K 10208160

[B72] Molosh A. I. Johnson P. L. Spence J. P. Arendt D. Federici L. M. Bernabe C. (2014). Social learning and amygdala disruptions in Nf1 mice are rescued by blocking p21-activated kinase. *Nat. Neurosci.* 17 1583–1590. 10.1038/nn.3822 25242307 PMC4213300

[B73] Myrick L. K. Deng P. Y. Hashimoto H. Oh Y. M. Cho Y. Poidevin M. J. (2015). Independent role for presynaptic FMRP revealed by an FMR1 missense mutation associated with intellectual disability and seizures. *Proc. Natl. Acad. Sci. U.S.A.* 112 949–956. 10.1073/pnas.1423094112 25561520 PMC4313821

[B74] Nakamura Y. Takahashi T. (2007). Developmental changes in potassium currents at the rat calyx of Held presynaptic terminal. *J. Physiol.* 581 1101–1112. 10.1113/jphysiol.2007.128702 17331991 PMC2170855

[B75] Natarajan R. Trivedi-Vyas D. Wairkar Y. P. (2013). Tuberous sclerosis complex regulates *Drosophila* neuromuscular junction growth via the TORC2/Akt pathway. *Hum. Mol. Genet.* 22 2010–2023. 10.1093/hmg/ddt053 23393158

[B76] Normand E. A. Crandall S. R. Thorn C. A. Murphy E. M. Voelcker B. Browning C. (2013). Temporal and mosaic Tsc1 deletion in the developing thalamus disrupts thalamocortical circuitry, neural function, and behavior. *Neuron* 78 895–909. 10.1016/j.neuron.2013.03.030 23664552 PMC4529124

[B77] Omrani A. Van Der Vaart T. Mientjes E. Van Woerden G. M. Hojjati M. R. Li K. W. (2015). HCN channels are a novel therapeutic target for cognitive dysfunction in Neurofibromatosis type 1. *Mol. Psychiatry* 10.1038/mp.2015.48 [Epub ahead of print]. PMC5603719 25917366

[B78] Osterweil E. K. Chuang S.-C. Chubykin A. A. Sidorov M. Bianchi R. Wong R. K. S. (2013). Lovastatin corrects excess protein synthesis and prevents epileptogenesis in a mouse model of fragile X syndrome. *Neuron* 77 243–250. 10.1016/j.neuron.2012.01.034 23352161 PMC3597444

[B79] Pieretti M. Zhang F. P. Fu Y. H. Warren S. T. Oostra B. A. Caskey C. T. (1991). Absence of expression of the FMR-1 gene in fragile X syndrome. *Cell* 66 817–822. 10.1016/0092-8674(91)90125-I 1878973

[B80] Rauen K. A. (2013). The RASopathies. *Annu. Rev. Genomics Hum. Genet.* 14 355–369. 10.1146/annurev-genom-091212-153523 23875798 PMC4115674

[B81] Renden R. Taschenberger H. Puente N. Rusakov D. A. Duvoisin R. Wang L. Y. (2005). Glutamate transporter studies reveal the pruning of metabotropic glutamate receptors and absence of AMPA receptor desensitization at mature calyx of Held synapses. *J. Neurosci.* 25 8482–8497. 10.1523/JNEUROSCI.1848-05.2005 16162930 PMC3375655

[B82] Rodriguez-Contreras A. Van Hoeve J. S. Habets R. L. P. Locher H. Borst J. G. G. (2008). Dynamic development of the calyx of Held synapse. *Proc. Natl. Acad. Sci. U.S.A.* 105 5603–5608. 10.1073/pnas.0801395105 18375766 PMC2291093

[B83] Rotschafer S. E. Razak K. A. (2014). Auditory processing in fragile X syndrome. *Front. Cell. Neurosci.* 8:19 10.3389/fncel.2014.00019 PMC3912505 24550778

[B84] Schuhmacher A. J. Guerra C. Sauzeau V. Cañamero M. Bustelo X. R. Barbacid M. (2008). A mouse model for Costello syndrome reveals an Ang II-mediated hypertensive condition. *J. Clin. Invest.* 118 2169–2179. 10.1172/JCI34385 18483625 PMC2381749

[B85] Schwenger D. B. Kuner T. (2010). Acute genetic perturbation of exocyst function in the rat calyx of Held impedes structural maturation, but spares synaptic transmission. *Eur. J. Neurosci.* 32 974–984. 10.1111/j.1460-9568.2010.07391.x 20849529

[B86] Seeger G. Yan L. Gärtner U. Huemmeke M. Barmashenko G. Mittmann T. (2004). Activation of Ras in neurons modifies synaptic vesicle docking and release. *Neuroreport* 15 2651–2654. 10.1097/00001756-200412030-00019 15570171

[B87] Seri S. Cerquiglini A. Pisani F. Curatolo P. (1999). Autism in tuberous sclerosis: evoked potential evidence for a deficit in auditory sensory processing. *Clin. Neurophysiol.* 110 1825–1830. 10.1016/S1388-2457(99)00137-6 10574297

[B88] Sharma A. Hoeffer C. A. Takayasu Y. Miyawaki T. Mcbride S. M. Klann E. (2010). Dysregulation of mTOR signaling in fragile X syndrome. *J. Neurosci.* 30 694–702. 10.1523/JNEUROSCI.3696-09.2010 20071534 PMC3665010

[B89] Shilyansky C. Karlsgodt K. H. Cummings D. M. Sidiropoulou K. Hardt M. James A. S. (2010a). Neurofibromin regulates corticostriatal inhibitory networks during working memory performance. *Proc. Natl. Acad. Sci. U.S.A.* 107 13141–13146. 10.1073/pnas.1004829107 20624961 PMC2919968

[B90] Shilyansky C. Lee Y. S. Silva A. J. (2010b). Molecular and cellular mechanisms of learning disabilities: a focus on NF1. *Annu. Rev. Neurosci.* 33 221–243. 10.1146/annurev-neuro-060909-153215 20345245 PMC3063104

[B91] Silva A. J. Frankland P. W. Marowitz Z. Friedman E. Laszlo G. S. Cioffi D. (1997). A mouse model for the learning and memory deficits associated with neurofibromatosis type I. *Nat. Genet.* 15 281–284. 10.1038/ng0397-281 9054942

[B92] Sommer I. Lingenhöhl K. Friauf E. (1993). Principal cells of the rat medial nucleus of the trapezoid body: an intracellular in vivo study of their physiology and morphology. *Exp. Brain Res.* 95 223–239. 10.1007/BF00229781 8224048

[B93] Spencer C. M. Alekseyenko O. Hamilton S. M. Thomas A. M. Serysheva E. Yuva-Paylor L. A. (2011). Modifying behavioral phenotypes in Fmr1KO mice: genetic background differences reveal autistic-like responses. *Autism Res.* 4 40–56. 10.1002/aur.168 21268289 PMC3059810

[B94] Stornetta R. L. Zhu J. J. (2011). Ras and Rap signaling in synaptic plasticity and mental disorders. *Neuroscientist* 17 54–78. 10.1177/1073858410365562 20431046 PMC3119507

[B95] Strumbos J. G. Brown M. R. Kronengold J. Polley D. B. Kaczmarek L. K. (2010). Fragile X mental retardation protein is required for rapid experience-dependent regulation of the potassium channel Kv3.1b. *J. Neurosci.* 30 10263–10271. 10.1523/JNEUROSCI.1125-10.2010 20685971 PMC3485078

[B96] Swiech L. Perycz M. Malik A. Jaworski J. (2008). Role of mTOR in physiology and pathology of the nervous system. *Biochim. Biophys. Acta* 1784 116–132. 10.1016/j.bbapap.2007.08.015 17913600

[B97] Tan M. L. Borst J. G. G. (2007). Comparison of responses of neurons in the mouse inferior colliculus to current injections, tones of different durations, and sinusoidal amplitude-modulated tones. *J. Neurophysiol.* 98 454–466. 10.1152/jn.00174.2007 17507505

[B98] Taschenberger H. von Gersdorff H. (2000). Fine-tuning an auditory synapse for speed and fidelity: developmental changes in presynaptic waveform, EPSC kinetics, and synaptic plasticity. *J. Neurosci.* 20 9162–9173. 11124994 10.1523/JNEUROSCI.20-24-09162.2000 PMC6773022

[B99] Tavazoie S. F. Alvarez V. A. Ridenour D. A. Kwiatkowski D. J. Sabatini B. L. (2005). Regulation of neuronal morphology and function by the tumor suppressors Tsc1 and Tsc2. *Nat. Neurosci.* 8 1727–1734. 10.1038/nn1566 16286931

[B100] Tolnai S. Englitz B. Scholbach J. Jost J. Rübsamen R. (2009). Spike transmission delay at the calyx of Held in vivo: rate dependence, phenomenological modeling, and relevance for sound localization. *J. Neurophysiol.* 102 1206–1217. 10.1152/jn.00275.2009 19515955

[B101] Troca-Marín J. A. Alves-Sampaio A. Montesinos M. L. (2012). Deregulated mTOR-mediated translation in intellectual disability. *Prog. Neurobiol.* 96 268–282. 10.1016/j.pneurobio.2012.01.005 22285767

[B102] Tsai P. T. Hull C. Chu Y. Greene-Colozzi E. Sadowski A. R. Leech J. M. (2012). Autistic-like behaviour and cerebellar dysfunction in Purkinje cell Tsc1 mutant mice. *Nature* 488 647–651. 10.1038/nature11310 22763451 PMC3615424

[B103] Varela J. A. Sen K. Gibson J. Fost J. Abbott L. F. Nelson S. B. (1997). A quantitative description of short-term plasticity at excitatory synapses in layer 2/3 of rat primary visual cortex. *J. Neurosci.* 17 7926–7940. 9315911 10.1523/JNEUROSCI.17-20-07926.1997 PMC6793910

[B104] Verkerk A. J. M. H. Pieretti M. Sutcliffe J. S. Fu Y. H. Kuhl D. P. A. Pizzuti A. (1991). Identification of a gene (FMR-1) containing a CGG repeat coincident with a breakpoint cluster region exhibiting length variation in fragile X syndrome. *Cell* 65 905–914. 10.1016/0092-8674(91)90397-H 1710175

[B105] Viosca J. Schuhmacher A. J. Guerra C. Barco A. (2009). Germline expression of H-*Ras*^G12V^ causes neurological deficits associated to Costello syndrome. *Genes Brain Behav.* 8 60–71. 10.1111/j.1601-183X.2008.00443.x 18823404

[B106] von der Brelie C. Waltereit R. Zhang L. Beck H. Kirschstein T. (2006). Impaired synaptic plasticity in a rat model of tuberous sclerosis. *Eur. J. Neurosci.* 23 686–692. 10.1111/j.1460-9568.2006.04594.x 16487150

[B107] Wang T. Rusu S. I. Hruskova B. Turecek R. Borst J. G. G. (2013). Modulation of synaptic depression of the calyx of Held synapse by GABA_B_ receptors and spontaneous activity. *J. Physiol.* 591 4877–4894. 10.1113/jphysiol.2013.256875 23940376 PMC3800460

[B108] Wang Y. Duan J. H. Hingtgen C. M. Nicol G. D. (2010). Augmented sodium currents contribute to the enhanced excitability of small diameter capsaicin-sensitive sensory neurons isolated from Nf1^+/-^ mice. *J. Neurophysiol.* 103 2085–2094. 10.1152/jn.01010.2009 20164394 PMC2853284

[B109] Wang Y. Nicol G. D. Clapp D. W. Hingtgen C. M. (2005). Sensory neurons from Nf1 haploinsufficient mice exhibit increased excitability. *J. Neurophysiol.* 94 3670–3676. 10.1152/jn.00489.2005 16093333

[B110] Weston M. C. Chen H. Swann J. W. (2014). Loss of mTOR repressors Tsc1 or Pten has divergent effects on excitatory and inhibitory synaptic transmission in single hippocampal neuron cultures. *Front. Mol. Neurosci.* 7:1 10.3389/fnmol.2014.00001 PMC3922082 24574959

[B111] Yamashita T. Ishikawa T. Takahashi T. (2003). Developmental increase in vesicular glutamate content does not cause saturation of AMPA receptors at the calyx of Held synapse. *J. Neurosci.* 23 3633–3638. 12736334 10.1523/JNEUROSCI.23-09-03633.2003 PMC6742176

[B112] Yang B. Desai R. Kaczmarek L. K. (2007). Slack and Slick K_Na_ channels regulate the accuracy of timing of auditory neurons. *J. Neurosci.* 27 2617–2627. 10.1523/JNEUROSCI.5308-06.2007 17344399 PMC6672517

[B113] Zhang Y. Q. Bailey A. M. Matthies H. J. G. Renden R. B. Smith M. A. Speese S. D. (2001). *Drosophila* fragile X-related gene regulates the MAP1B homolog Futsch to control synaptic structure and function. *Cell* 107 591–603. 10.1016/S0092-8674(01)00589-X 11733059

[B114] Zhu J. J. Qin Y. Zhao M. Van Aelst L. Malinow R. (2002). Ras and Rap control AMPA receptor trafficking during synaptic plasticity. *Cell* 110 443–455. 10.1016/S0092-8674(02)00897-8 12202034

