# Supplementary material for: In vivo synaptic transmission and morphology in mouse models of Tuberous sclerosis, Fragile X syndrome, Neurofibromatosis type 1, and Costello syndrome
Source: Front Cell Neurosci. 2015 Jul 3;9:234. doi: 10.3389/fncel.2015.00234 (PMC4490249; doi:10.3389/fncel.2015.00234)
Supplement: Supplementary file 1 [file Data_Sheet_1.PDF]

Supplementary Material

In vivo synaptic transmission and morphology in mouse models of Tuberous sclerosis, Fragile X syndrome, Neurofibromatosis type 1 and Costello syndrome

Tiantian Wang[1], Laura de Kok[1], Rob Willemsen[2], Ype Elgersma[1,3], J. Gerard G. Borst[1*]

[1]Department of Neuroscience, Erasmus MC, University Medical Center Rotterdam, Rotterdam, The Netherlands.
[2]Department of Clinical Genetics, Erasmus MC, University Medical Center Rotterdam, Rotterdam, The Netherlands.
[3]ENCORE Expertise Center for Neurodevelopmental disorders, Erasmus MC, University Medical Center Rotterdam, Rotterdam, The Netherlands

*** Correspondence:** Corresponding Author, Dr. Gerard Borst, Department of Neuroscience, Erasmus MC, University Medical Center Rotterdam, Dr. Molewaterplein 50, 3015 GE Rotterdam, The Netherlands.
g.borst@erasmusmc.nl

Supplementary Figures

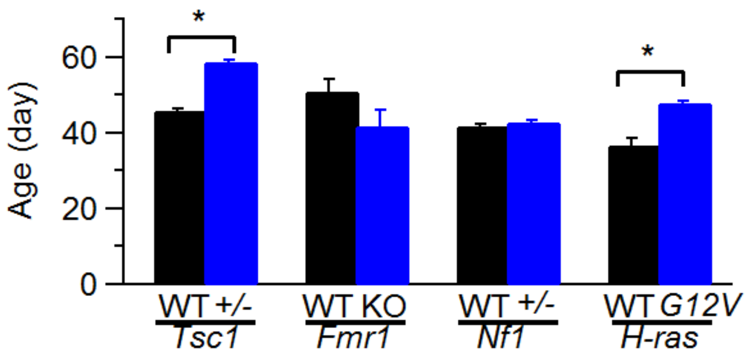

Figure S1. Average ages for morphological studies of the four mutant lines and their WT controls. Average age between WT and mutant for the morphological studies were 46 ± 0.4 (n = 13) and 59 ± 0.4 days (n = 14; $p < 0.01$) for WT and *Tsc1*$^{+/-}$ mice, respectively, 51 ± 3 (n = 19 calyces) and 42 ± 4 days (n = 29; $p = 0.08$) for WT and *Fmr1* KO mice, respectively, 42 ± 0.3 (n = 10) and 43 ± 0.3 days (n = 13; $p = 0.05$) for WT and *Nf1*$^{+/-}$ mice, respectively, 37 ± 2 (n = 13), and 49 ± 0.5 (n = 10; $p < 0.01$) for WT and *H-ras*^{G12V} mice, respectively.

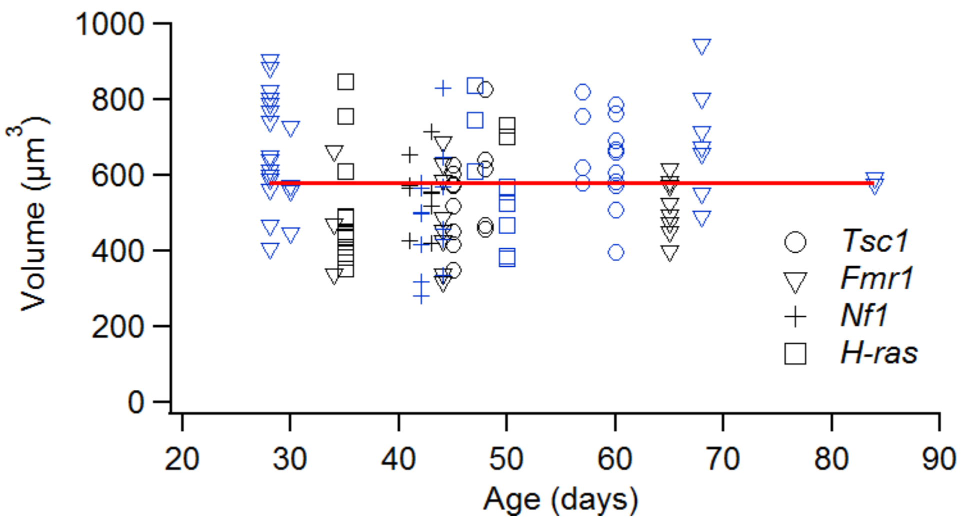

Figure S2. Relation between calyceal volume and age of the mouse. Black symbols indicate WT, blue symbols indicate mutant. Red line is the result of the linear regression (r = 0.032; p = 0.73).

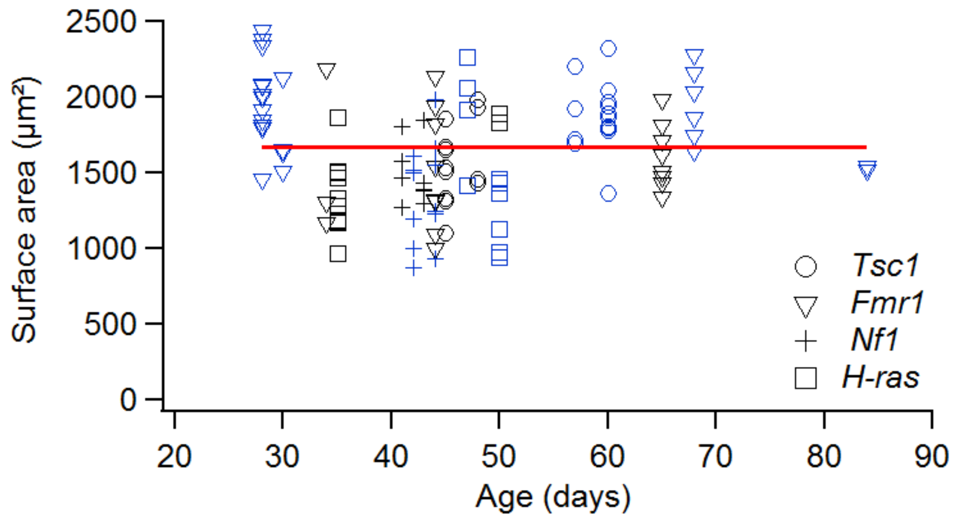

Figure S3. Relation between calyceal surface area and age of the mouse. Black symbols indicate WT, and blue ones indicate mutant. Red line is the result of the linear regression (r = 0.00; p = 0.99).

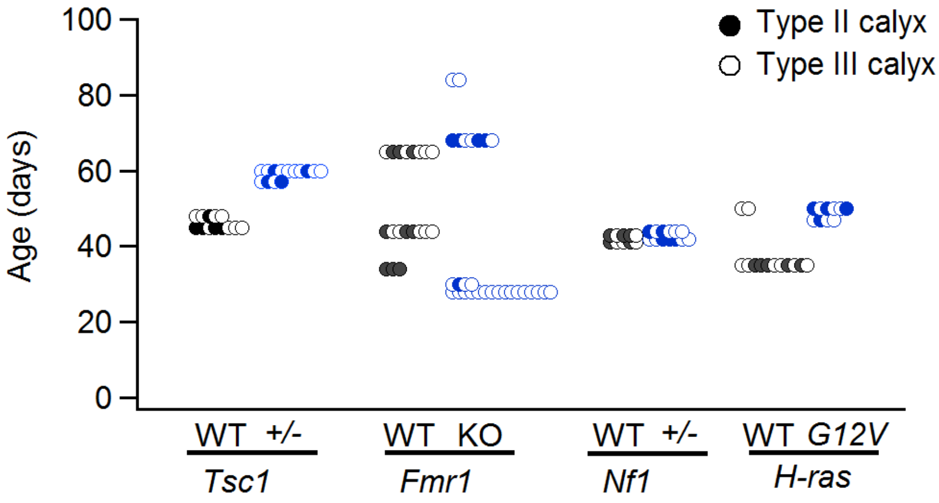

Figure S4. Comparison of ages of all the cells from four lines in the morphological study. Each circle represent one cell. Black indicates WT; blue indicates mutant. Closed circles indicate type II calyces (6-15 boutons), and open circles indicate type III calyces (>15 boutons). This figure illustrates the lack of age dependence of the classification in Type II or Type III and the lack of difference in age between WT and control.

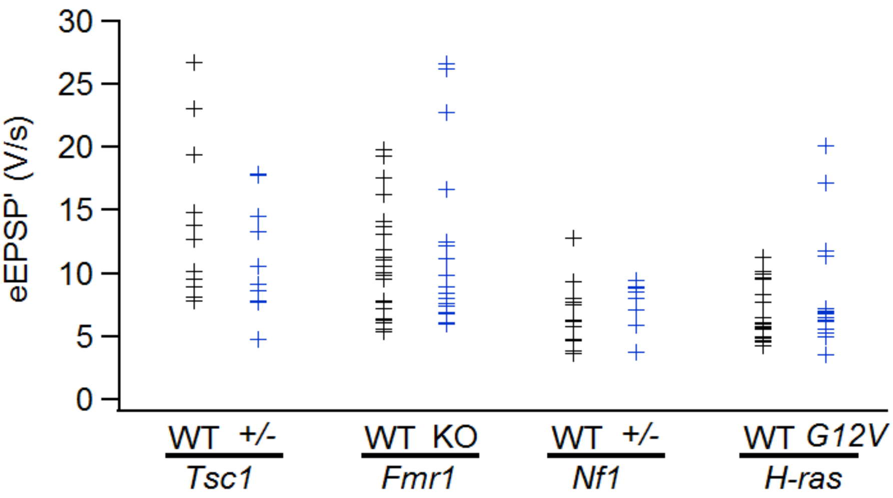

Figure S5. Absolute sizes of eEPSP' of four lines. Black symbols indicate WT, and blue ones indicate the mutant. T-test showed no significant difference ($p > 0.05$ for all four lines). The average eEPSP' sizes of four lines were: 13.8 ± 0.1 V/s for *Tsc1* WT, $n = 12$ vs. 11.2 ± 1.3 V/s for *Tsc1*$^{+/-}$, $n = 10$; $p = 0.29$; 10.9 ± 0.8 V/s for *Fmr1* WT, $n = 26$ vs. 11.9 ± 1.6 V/s for *Fmr1* KO, $n = 18$; $p = 0.56$; 6.8 ± 0.6 V/s for *Nf1* WT, $n = 14$ vs. 7.7 ± 0.6 V/s for *Nf1*$^{+/-}$, $n = 9$; $p = 0.35$; 7.0 ± 0.5 V/s for *H-ras* WT, $n = 20$ vs. 8.4 ± 1.2 V/s for *H-ras*^{G12V}, $n = 15$; $p = 0.25$.
